# Strand-specific RNA sequencing in *Plasmodium falciparum* malaria identifies developmentally regulated long non-coding RNA and circular RNA

**DOI:** 10.1186/s12864-015-1603-4

**Published:** 2015-06-13

**Authors:** Kate M Broadbent, Jill C Broadbent, Ulf Ribacke, Dyann Wirth, John L Rinn, Pardis C Sabeti

**Affiliations:** Department of Systems Biology, Harvard Medical School, Boston, Massachusetts USA; Broad Institute, Cambridge, Massachusetts USA; FAS Center for Systems Biology, Harvard University, Cambridge, Massachusetts USA; Department of Organismic and Evolutionary Biology, Harvard University, Cambridge, Massachusetts USA; Department of Immunology and Infectious Diseases, Harvard School of Public Health, Boston, Massachusetts USA; Department of Cell and Molecular Biology, BMC, Uppsala University, Uppsala, Sweden; Department of Stem Cell and Regenerative Biology, Harvard University, Cambridge, Massachusetts USA

**Keywords:** RNA sequencing, Non-coding RNA, lncRNA, Antisense RNA, circRNA, microRNA, Malaria, *Plasmodium*, Transcriptome, Gene regulation, Extreme genome, PfGDV1

## Abstract

**Background:**

The human malaria parasite *Plasmodium falciparum* has a complex and multi-stage life cycle that requires extensive and precise gene regulation to allow invasion and hijacking of host cells, transmission, and immune escape. To date, the regulatory elements orchestrating these critical parasite processes remain largely unknown. Yet it is becoming increasingly clear that long non-coding RNAs (lncRNAs) could represent a missing regulatory layer across a broad range of organisms.

**Results:**

To investigate the regulatory capacity of lncRNA in *P. falciparum*, we harvested fifteen samples from two time-courses. Our sample set profiled 56 h of *P. falciparum* blood stage development. We then developed and validated strand-specific, non-polyA-selected RNA sequencing methods, and pursued the first assembly of *P. falciparum* strand-specific transcript structures from RNA sequencing data. This approach enabled the annotation of over one thousand lncRNA transcript models and their comprehensive global analysis: coding prediction, periodicity, stage-specificity, correlation, GC content, length, location relative to annotated transcripts, and splicing. We validated the complete splicing structure of three lncRNAs with compelling properties. Non-polyA-selected deep sequencing also enabled the prediction of hundreds of intriguing *P. falciparum* circular RNAs, six of which we validated experimentally.

**Conclusions:**

We found that a subset of lncRNAs, including all subtelomeric lncRNAs, strongly peaked in expression during invasion. By contrast, antisense transcript levels significantly dropped during invasion. As compared to neighboring mRNAs, the expression of antisense-sense pairs was significantly anti-correlated during blood stage development, indicating transcriptional interference. We also validated that *P. falciparum* produces circRNAs, which is notable given the lack of RNA interference in the organism, and discovered that a highly expressed, five-exon antisense RNA is poised to regulate *P. falciparum* gametocyte development 1 (*PfGDV1*), a gene required for early sexual commitment events.

**Electronic supplementary material:**

The online version of this article (doi:10.1186/s12864-015-1603-4) contains supplementary material, which is available to authorized users.

## Background

*Plasmodium falciparum* is the most deadly human malaria parasite, notorious for its immense disease burden, ability to persist in individuals for months if not longer, and rapid development of resistance to all currently available treatments [1–4]. The symptomatic characteristics of acute *P. falciparum* malaria infection correspond to cycles of red blood cell (RBC) rupture, as merozoite parasites invade RBCs, asexually replicate into 8–36 new daughter merozoites, egress from the RBCs, and repeat the process every 48 h [5–8]. This process can be readily modeled in the lab, in contrast to the sexual stage required for transmission, which takes 8–12 days in human RBCs and then an additional 8–15 days in mosquitoes [9, 10]. Due to the clinical symptoms associated with the asexual blood stage and the relative ease of obtaining samples, the vast majority of current anti-malarial compounds and research programs target this stage of the parasite life cycle [11]. However, the idea of targeting both the symptomatic and transmissible parasite form is garnering increased public attention, making research on sexual stage commitment and sexual development a priority as well [11–13].

The first *P. falciparum* genome sequence was published in 2002 [14]. Our understanding of malaria biology has advanced considerably since this milestone, largely due to genome-wide studies [15, 16]. Early transcriptome studies found that key *P. falciparum* protein-coding genes are typically transcribed only once per blood stage, ‘just-in-time’ for translation and function [17, 18]. Subsequently, global ribosome profiling and proteome studies revealed significant post-transcriptional regulation and a unique histone code involving at least 44 histone post-translational modifications and four novel histone variants [19–22]. Additionally, paired transcriptome-epigenome studies found dynamic chromatin remodeling and clonally variant gene expression (CVGE) patterns during blood stage development [23–26]. Independent studies have confirmed a heritable epigenetic layer to monoallelic expression of the 60-member *P. falciparum* Erythrocyte Membrane Protein 1 (PfEMP1*)*-encoding *var* gene family, as well as heritable epigenetic regulation of genes involved in invasion and nutrient uptake [27–33].

While it has become increasingly clear over the past decade that the *P. falciparum* genome is tightly regulated, the regulatory elements themselves are still largely uncharacterized [34, 35]. For example, it is not mechanistically clear how the parasite transcriptionally silences, activates, or switches PfEMP1-encoding *var* genes to evade the human immune system, or how the parasite switches from asexual to sexual development [36, 37]. Few sequence-specific transcription factors have been identified, and *P. falciparum* does not encode identifiable microRNAs, microRNA processing machinery, or RNA-induced silencing complex (RISC) components [38–40]. With the absence of many known transcription factors and the canonical RNA interference pathway, master regulatory elements orchestrating immune escape, invasion, transmission, and other critical parasite processes remain to be discovered.

We hypothesized that further study of *P. falciparum* long non-coding RNA (lncRNA) may provide missing insights into *P. falciparum* transcriptional, post-transcriptional, and chromatin state control. Encouragingly, previous survey studies have demonstrated non-coding transcription in *P. falciparum* [41–46], and a growing body of evidence supports the crucial regulatory roles of lncRNAs in humans and model organisms [47, 48]. For example, it has been shown that lncRNAs coordinate X chromosome inactivation in female mammalian cells, flowering time in plants, and gametogenesis in budding yeast [49–54]. A handful of circRNAs have been recently shown to function as microRNA sponges as well [55, 56]. While prior work has suggested the transcription of intergenic, antisense, and even circular RNA (circRNA) in *P. falciparum*, lncRNA transcript models have not been defined and transcript properties have not been generalized on a broad scale [41–45].

In this study, we assemble 660 intergenic lncRNA and 474 antisense transcript structures from strand-specific *P. falciparum* RNA sequencing reads (202 antisense loci are entirely novel), compile a comprehensive catalog of transcript properties, summarize global trends, and experimentally validate the splicing structure of three *P. falciparum* lncRNAs with exceptional properties. We also predict the transcription of hundreds of novel *P. falciparum* circRNA candidates (6/9 experimental confirmation rate), and search for human microRNA binding sites across *P. falciparum* coding sequences. To our knowledge, the latter analysis has not been reported previously, nor has a role for human microRNA binding interactions within *P. falciparum* transcripts. On the other hand, LaMonte et al. and others have shown that human microRNAs do indeed translocate from the red blood cell into *P. falciparum* [57, 58]*.*

Although many studies, including our own, have provided insights into the *P. falciparum* non-coding transcriptome, an in depth strand-specific catalog was critically needed to accelerate hypothesis generation and experimental testing [41–44]. As an example of the novel insights that this work provides, we have identified that lncRNA and mRNA expression dynamics differ during parasite invasion, have found evidence that antisense-sense transcriptional interference is prevalent during the blood stage, and have contributed the initial characterization and structural validation of a highly expressed, non-coding counterpart to *P. falciparum* gametocyte development 1 (*PfGDV1*).

## Results

### Strand-specific RNA sequencing of biological replicate blood stage time courses

**Fig. 1:**
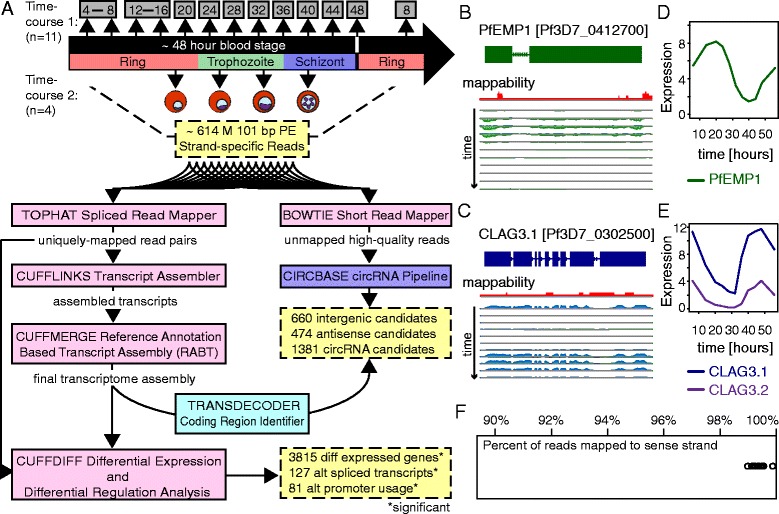
Overview of *P. falciparum* RNA sequencing sample set, computational pipeline, and read alignment metrics. **(A)** We harvested total RNA from two independent *P. falciparum* blood stage time courses, including a 56-hour time course consisting of eleven samples. We combined samples harvested 4 and 8 hpi at equal ratios (further referred to as T6). Similarly, we combined samples harvested 12 and 16 hpi at equal ratios (further referred to as T14). We harvested four additional samples from a second time course approximately 4 h before and after gross stage transitions. Thus these samples correspond to the late ring, early trophozoite, late trophozoite, and early schizont stages, respectively. In total, we sequenced fifteen strand-specific RNA sequencing (RNA-seq) libraries on an Illumina Hiseq 2000 machine. Illumina sequencing yielded approximately 614 million 101-bp paired-end reads. We analyzed reads using the Tuxedo suite (Bowtie, TopHat, Cufflinks, Cuffmerge, and Cuffdiff) and according to the circBase circRNA discovery pipeline [85]. Using this approach, we identified 660 intergenic lncRNA (647 unique loci), 474 antisense RNA (467 unique loci), and 1381 circRNA candidates. Additionally, 3815 genes, 127 transcripts, and 81 promoters reached statistical significance in terms of differential expression, alternative splicing, and alternative promoter usage, respectively. **(B)**/**(C)** Normalized read alignment tracks across a PfEMP1-encoding *var* gene [PlasmoDB:*Pf3D7_0412700*] and the *CLAG3.1* gene [PlasmoDB:*Pf3D7_0302500*] indicated that these challenging loci could generally be (perfectly and uniquely) mapped. Annotated gene models are shown in dark green and dark blue. Reads from each 56-hour time course sample mapping to the (−) strand are shown below each horizontal axis in light green, while reads mapping to the (+) strand are shown above each horizontal axis in light blue. Uniqueness of 100mers is plotted in red as a mappability track, where the baseline represents a score of one, or uniquely mapping. **(D)**/**(E)** Plotting the expression during the 56-hour time course of the dominant PfEMP1-encoding *var* gene [PlasmoDB:*Pf3D7_0412700*] and both the *CLAG3.1* [PlasmoDB:*Pf3D7_0302500*] and *CLAG3.2* [PlasmoDB:*Pf3D7_*0502200] genes showed, respectively, that *var* gene expression peaked during the ring stage, whereas *CLAG3.1* and *CLAG3.2* expression peaked during the schizont stage. Moreover, as *CLAG3* genes are mutually exclusively expressed [27, 28], we found that that the bulk of our parasites transcribed only the *CLAG3.1* gene. Expression is plotted in units of log2(FPKM + 1). **(F)** The percent of reads in each library mapping to annotated transcripts in the proper orientation (per reads mapping to annotated transcripts) ranged from 98.92 % to 99.81 %. The average calculated from both reads is reported

To investigate *P. falciparum* lncRNA transcription, we harvested fifteen blood stage samples from two biological replicate time courses [Fig. 1A]. The first time course comprised eleven samples harvested over 56 h from a tightly synchronized *P. falciparum* 3D7 parasite population: 6, 14, 20, 24, 28, 32, 36, 40, 44, 48, and 56 h post-infection (hpi). As the asexual blood stage is an approximately 48-hour cycle, this sample set allowed us to profile gene expression during the critical process of RBC rupture and parasite invasion. The second time course comprised four samples harvested in synchronous *P. falciparum* 3D7 parasites approximately four hours before and after the ring to trophozoite and trophozoite to schizont morphological stage transitions, which occur during the blood stage at 24 hpi and 36 hpi, respectively.

Given the high AT content and dense genic structure of the *P. falciparum* genome, we then extensively optimized RNA sequencing procedures, both experimental and computational, in order to derive a high-quality *P. falciparum* transcriptome. In terms of experimental optimization, we tested numerous variables and pursued technical developments shown to reduce sequence-based bias in DNA sequencing libraries and to improve strand-specificity in RNA sequencing libraries [59–65]. Subsequently, we established a library preparation protocol that uses multiple DNase treatments to remove genomic DNA, Ribo-Zero beads to remove ribosomal RNA, the dUTP method with Actinomycin D to preserve strand specificity, and the KAPA HiFi polymerase to amplify libraries in real-time for the minimum number of cycles necessary [See Methods, and Additional files 1 and 2 for further details].

After harvesting samples from the two time courses and developing and validating our strand-specific library preparation protocol, we prepared libraries from the fifteen blood stage samples in parallel, and sequenced libraries on two lanes of an Illumina Hiseq 2000 machine. Illumina sequencing yielded 614 million 101 base-pair (bp) paired-end reads in total, with sequencing depth ranging from 20 to 30 million (perfectly and uniquely) alignable reads per sample. We noted high base quality scores and no significant adapter contamination [Additional files 3, 4 and 5].

### Aligning and benchmarking sequences

We took a conservative approach to read alignment, requiring read pairs to map perfectly and uniquely to the *P. falciparum* 3D7 reference genome. In support of this, we determined that 96.53 % of all possible 100mers in the *P. falciparum* genome are unique. In addition, we tested our ability to map read pairs across repeated gene families, such as the PfEMP1-encoding *var* gene family and the two Cytoadherence-Linked Asexual Gene 3 (*CLAG3*) loci, which we calculated share 96.4 % sequence similarity. Specifically, we visualized a lower bound to mappability across these repeated loci by plotting the uniqueness of 100mers as a mappability track. Fig. 1B and C show the mappability track (in red) compared to strand-specific read coverage across a PfEMP1-encoding *var* gene [PlasmoDB:*Pf3D7_0412700*] and the *CLAG3.1* gene [PlasmoDB:*Pf3D7_0302500*], respectively. Fig. 1C and D plot the expression profiles of these genes, as well as the *CLAG3.2* gene [PlasmoDB:*Pf3D7_0302200*]. Using this stringent approach and paired-end information, we were able to uniquely map read pairs to these repeated loci, including through short stretches of non-unique sequence.

After conservatively aligning reads using TopHat [66], we assessed data quality following the RNA sequencing benchmarking metrics put forth by DeLuca et al. and Wang et al. [67, 68]. We calculated the strand-specificity, coefficient of variation, duplication rate, gap rate, ribosomal RNA rate, exonic rate, insert size, and GC content of each aligned set of reads [Additional file 4]. Importantly, we found that greater than 98.92 % of reads mapped to the reference strand in the expected orientation in each sample [Fig. 1F]. This result was on par with yeast strand-specific sequencing libraries, and confirmed that our data was highly strand-specific [60]. We also found an average coefficient of variation (CV) of between 0.23 and 0.33 across the top 2000 expressed genes (or roughly top 50 % of expressed genes) in each sample [Additional file 4]. These CV values were lower than the lowest CV value reported in the benchmarking study referenced above (0.54), indicating more even read coverage in our samples [60]. Taken together, the rigorous examination of our data quality demonstrated that it was comparable to the state-of-the-art in model organisms.

### Benchmarking time courses

**Fig. 2:**
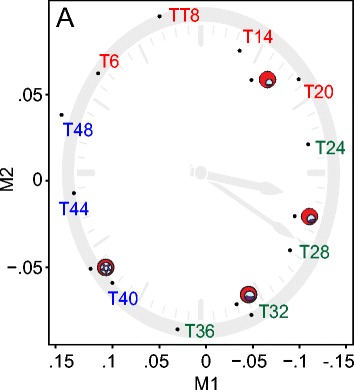
Multidimensional scaling and Gene Ontology confirm expected *P. falciparum* blood stage expression patterns. The MDS plot of sample profiles embedded samples around a circle. Traversing the circle, we found that samples progressed through the approximately 48-hour *P. falciparum* blood stage according to their time and morphology labels as expected. The 56-hour time course samples are labeled in red, green, and blue, with red corresponding to samples harvested within the predicted ring stage, green corresponding to samples harvested within the predicted trophozoite stage, and blue corresponding to samples harvested within the predicted schizont stage. The morphology-based labels correspond to the late ring, early trophozoite, late trophozoite, and early schizont stages, respectively

Comparing samples between two independent time courses is a known challenge in the field, and can be confounded by experimental factors such as culture conditions [26, 69, 70]. We thus developed a computational solution that leverages multidimensional scaling (MDS) to assess stage similarities on a transcriptome-wide scale. While MDS has not previously been used for *P. falciparum* sample comparisons, its utility has been demonstrated in humans and in model organisms such as yeast, especially when periodicity is expected [71, 72]. MDS analysis using sample profiles from both time courses revealed the cyclical nature of the *P. falciparum* blood stage, with samples progressing in time around an approximately 48-hour clock [Fig. 2]. This analysis also confirmed that the four morphology-based samples corresponded to the 56-hour, high-resolution time course samples at expected intervals.

As a complementary analysis, we classified 1632 ring-specific, 1378 trophozoite-specific, and 1274 schizont-specific genes according to their maximal expression time-point. We then computed GO term enrichment on these stage-specific gene sets [73]. Ring-, trophozoite-, and schizont-specific GO terms were specific to host cell adhesion processes, metabolic processes, and protein catabolic processes, respectively, with DNA replication spanning both trophozoite- and schizont-specific GO terms [Additional files 6 and 7]. These GO terms were highly consistent with our current understanding of *P. falciparum* biology. Taken together, MDS paired with global GO term enrichment analysis validated the biological integrity of our time course samples.

### Transcript assembly

We next set out to assemble *P. falciparum* transcript structures, with or without the assistance of annotated transcript models, and to assess assembly performance using either Cufflinks or genome-guided Trinity [74–76] [Fig. 1A]. Specifically, we were looking for high contiguity (a high rate of annotated transcripts being spanned by one assembled transcript over at least 90 % of the annotated transcript exonic length), low chimerism (a low rate of assembled transcripts spanning more than one annotated transcript), and for the final assembly to be manageable and high-confidence [77, 78]. Importantly, our calculations conservatively assumed that all of the chimeric predictions represent assembly artifacts. However, it is worth noting that some portion may represent bona fide products of the spliceosome machinery [Additional file 8].

Based on its performance features, we chose to further explore the high-confidence Cufflinks transcripts (at least 50 supporting read fragments in at least one sample). However, it may be possible to filter the genome-guided Trinity results based on read support or expression level to yield a more manageable *P. falciparum* transcriptome assembly [Table 1, Additional file 9]. Using Cufflinks without the assistance of annotation, we found that 81.5 % of annotated transcripts had assembled transcripts contiguously spanning them, while only 6.6 % of assembled transcripts were chimeric [Table 1, Additional file 10]. With the assistance of annotation, the chimerism rate dropped to 4.5 % and the contiguity rate naturally rose to 100 % [Table 1, Additional file 11]. For reference, Lu et al. reported a chimerism rate of 6 %, 14 %, and 22 % in human, mouse, and yeast Cufflinks assemblies, respectively [77]. We thus considered the proportion of chimeric transcripts in our Cufflinks assemblies to be acceptably low.

**Table 1:** Comparative assessment of P. falciparum transcriptome assembly highlights the performance of Cufflinks RABT

	Contiguity	Chimerism	Total number of transcripts	Number of intergenic transcripts	Number of antisense transcripts
*PlasmoDBv10.0*			5,777		
Cufflinks	81.5 %	6.6 %	7,065	660	479
Cufflinks RABT	100 %	4.5 %	9,434	660	474
Genome-guided Trinity	57.1 %	1.3 %	43,816	8,260	11,070
Genome-guided Trinity RABT	100 %	1 %	21,182	5,839	7,234

We compared the contiguity, chimerism, and feature counts of Cufflinks versus Genome-guided Trinity transcriptome assembly, with or without the assistance of annotation. Cufflinks incorporating reference annotation based transcriptome assembly (RABT) provided the optimal *P. falciparum* transcriptome.

Contiguity is the rate of annotated transcripts covered by one assembled transcript over at least 90 % of the annotated transcript exonic length in the correct orientation. Chimerism is the rate of assembled transcripts that span more than one annotated transcript in the correct orientation. Total number of transcripts, number of intergenic transcripts, and number of antisense transcripts correspond to the total number of assembled transcripts, the number of assembled transcripts predicted between *PlasmoDBv10.0* annotations, and the number of assembled transcripts predicted antisense to *PlasmoDBv10.0* annotations. In total, 5,777 transcripts are annotated in *PlasmoDBv10.0*

*RABT = Reference annotation based transcript assembly

To further benchmark Cufflinks assembly performance in *P. falciparum*, we compared the expression properties, GC content, and length of Cufflinks-assembled transcripts to those of previous annotations. Towards this end, we paired 5727 and 7736 assembled transcripts with *PlasmoDBv10.0* annotated transcripts in the unassisted and assisted Cufflinks assemblies, respectively. We then calculated the correlation between paired expression profiles, finding a median correlation of 0.98 and 0.99 for unassisted and assisted transcripts, respectively. This led us to conclude that analyzing assembled transcript expression profiles was essentially interchangeable with analyzing annotated transcript expression profiles. We did, however, note a shift towards lower FPKM (fragments per kilobase of exon per million fragments mapped) expression level and lower GC content for assembled transcripts. This was largely because assembled transcripts included unannotated, likely untranslated regions (UTRs) with reduced read support and GC content as compared to coding regions [Additional files 12 and 13]. We selected the annotation-assisted Cufflinks transcriptome for further analyses, unless otherwise noted, as it represented the most complete *P. falciparum* transcriptome.

**Fig. 3:**
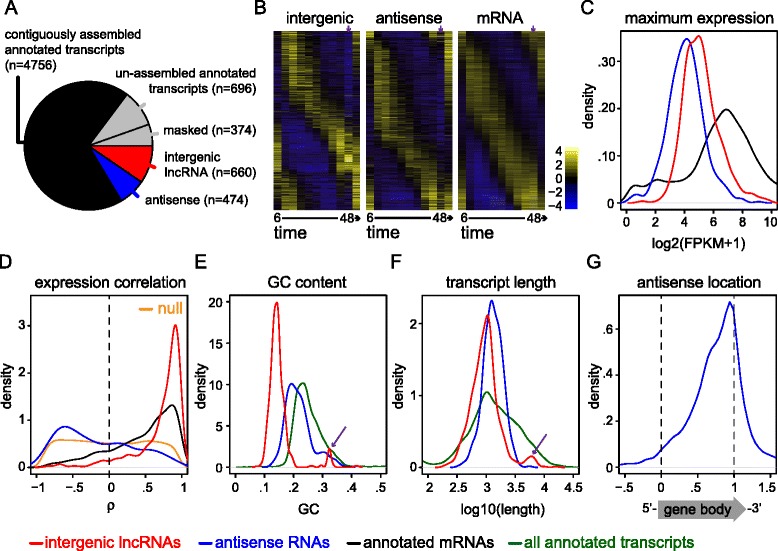
Characterization of 1134 unannotated *P. falciparum* lncRNAs reveals global trends as well as intriguing outliers. **(A)** Without annotation assistance, at least 4707 out of 5777 (81.5 %) annotated transcripts could be contiguously assembled in our blood stage samples. 696 annotated transcripts could not be contiguously assembled, and we excluded 374 short and/or structural RNAs from assembly. Given this high reassembly rate of known transcripts, it is possible that the 660 intergenic lncRNAs and 474 antisense RNAs described here represent the majority of lncRNAs transcribed in *P. falciparum*. **(B)** Comparative inspection of non-clustering heatmaps showed that predicted lncRNAs were developmentally regulated in a similar periodic fashion to annotated mRNAs. However, it was also apparent that a subset of lncRNAs strongly peaked in expression during parasite invasion, and that there was a paucity of antisense transcript levels during parasite invasion. The 48 hpi invasion time-point is indicated with purple arrows. Transcripts are ordered by their angular position in the MDS plot of transcript expression profiles, and samples are ordered by time. Mean-centered expression is in units of log2(FPKM + 1). **(C)** The distribution of maximum expression levels for each transcript class suggested that both intergenic lncRNAs (red) and antisense RNAs (blue) were robustly expressed, albeit they typically reached lower maximum expression levels than annotated mRNAs (black). **(D)** Pearson correlation during the 56-hour time course between 50,000 random mRNA gene pairs (orange) as compared to 5251 mRNA-neighboring gene pairs (black), 498 intergenic lncRNA-neighboring gene pairs (red), and 445 antisense-sense gene pairs (blue). To be consistent, we defined the neighboring gene used in both the mRNA and intergenic lncRNA pairings as the more correlated neighboring mRNA. **(E)** The distribution of GC content for each transcript class indicated that intergenic lncRNAs (red) and antisense RNAs (blue) typically had lower GC content than annotated transcripts (green), though a handful of intergenic lncRNAs had unusually high GC content (purple arrow). **(F)** The distribution of transcript length for each transcript class showed that intergenic lncRNAs (red) and antisense RNAs (blue) were comparable in length to annotated transcripts (green), with the average of each class being longer than 1 kb. Markedly long intergenic lncRNAs (>4 kb) are indicated with a purple arrow. **(G)** Plotting the normalized distribution of antisense RNAs relative to annotated gene bodies revealed a 3’ tail-to-tail bias

In sum, annotation-assisted Cufflinks assembly predicted 9434 transcripts, including 660 unannotated intergenic transcripts (647 unique loci) and 474 antisense transcripts (467 unique loci; 202 novel loci) [Figs. 1A and 3A, and Additional files 14, 15, 16 and 17]. The 467 antisense loci overlapped 462 annotated genes in an approximately 1:1 ratio. This encompassed transcription of at least 73 % of the *P. falciparum* genome, a 13 % increase compared to annotation alone, and included the prediction of high-confidence antisense transcription from 8 % of annotated genes. Annotation-assisted Cufflinks assembly also predicted 2134 novel splice-junctions [Additional file 17]. On the other hand, Cufflinks assembly without annotation rediscovered 6918 out of 8537 annotated splice-junctions (81 %) and, as noted above, predicted contiguous transcripts spanning 4707 out of 5777 annotated transcripts (81.5 %) [Fig. 3A, Additional file 17].

### Coding region identification

To determine the coding potential of the 1134 previously unannotated transcripts, we used TransDecoder and found that at least 98.5 % represented bona fide non-coding RNAs [75]. TransDecoder predicted putative coding regions in 5213 out of the 5229 (99.7 %) possible protein-coding transcripts [Additional file 18], but in just seven out of the 660 intergenic transcripts (1.1 %) and eleven out of the 474 antisense transcripts (2.3 %) [Fig. 1A]. These proportions of putative coding regions in our candidate lncRNA sets did not significantly differ from the proportions that TransDecoder predicted in random regions (97 out of 6600 random intergenic regions, Fisher’s exact test, *p*-value = .493; 57 out of 4740 random antisense regions, Fisher’s exact test, *p*-value = .053). Moreover, we did not find precedent for overlapping genes in *P. falciparum* [14]. Given this body of data and the small proportion that the ambiguous transcripts represented in their respective data sets, we retained but noted these transcripts for further investigation [column Q in Additional files 15 and 16].

### LncRNA transcript properties

After ensuring data integrity, including validating the non-coding nature of unannotated transcripts, we set out to characterize lncRNA transcript properties. Towards this end, we first compared the expression periodicity of lncRNA transcripts to that of annotated mRNA transcripts, as stage-specific expression is likely to correlate with function. Indeed, when we visualized the expression of each transcript class in a non-clustering heatmap, we found a similar pattern of developmental regulation for both lncRNAs and mRNAs [Fig. 3B], although lncRNAs typically reached lower maximum expression levels than mRNAs [Table 2, Fig. 3C]. Motif prediction in the putative promoter regions (1 kb upstream) of both lncRNAs and mRNAs also returned many motifs in common [Additional file 19] [79]. Taken together, this global analysis revealed the remarkable similarity between lncRNA and mRNA expression cascades during blood stage development, and suggested stage-specific roles for *P. falciparum* lncRNAs.

**Table 2:** Global properties of *P. falciparum* lncRNAs include reduced expression, length, GC content, and splicing as compared to annotated transcripts

	LncRNA	Antisense	Annotated
Average of maximum FPKMs	56	25	469
Average of average FPKMs	18	8	164
Average Length	1218	1413	2197
Average GC content	15.0 %	21.8 %	25.4 %
Single exon rate	93.5 %	89.5 %	47.8 %
Maximum exon count	3	5	34

We calculated the average of maximum FPKMs and average of average FPKMs across each transcript class during the 56-hour time course. Average length and average GC content reflect exonic sequence only. Annotated transcript properties refer to *PlasmoDBv10.0* transcript models

This visual approach also highlighted two distinct lncRNA expression profile deviations during RBC rupture and parasite invasion [purple arrows in Fig. 3B]. Upon close inspection of the intergenic lncRNA expression profiles shown in Fig. 3B, we noted that a subset of intergenic lncRNAs strongly peaked in expression during the 48 hpi invasion time-point. We found that this subset included all members of the family of telomere-associated lncRNA-TAREs that we previously identified [41]. Second, upon close inspection of the antisense RNA expression profiles shown in Fig. 3B, we noted a paucity of antisense transcript levels during parasite invasion. In fact, we calculated that out of the 35 % of antisense RNAs (166) increasing in expression between 36–44 hpi, 72 % dropped in expression during parasite invasion and then increased in expression afterwards. A similar percentage of annotated mRNA transcripts (27 % or 1435) increased in expression between 36–44 hpi, but only 19 % exhibited the invasion-specific expression drop (Fisher’s exact test, *p*-value < .0001).

We next investigated the correlation properties of *P. falciparum* lncRNAs and annotated mRNAs, as positive or negative correlation between lncRNAs and neighboring genes may indicate a regulatory relationship [51, 80, 81]. Specifically, we compared the expression correlation between randomly sampled mRNAs (location-independent null) to that of the following location-dependent gene pairs: (1) annotated mRNAs and their more correlated neighboring mRNA, (2) intergenic lncRNAs and their more correlated neighboring mRNA, and (3) sense-antisense partners. We observed significantly more positively correlated intergenic lncRNA-neighbor pairs and mRNA-neighbor pairs than random mRNA pairs (Wilcoxon rank sum *p*-value < 2.2e-16 in both cases) [Fig. 3D]. On the other hand, we found that sense-antisense partners exhibited an entirely different expression correlation trend. Namely, we observed significantly more negatively (or anti-) correlated sense-antisense pairs than random mRNA pairs (Wilcoxon rank sum *p*-value = 3.834e-11) [Fig. 3D].

Interestingly, we found that intergenic lncRNA-neighbor pairs were significantly more positively correlated than mRNA-neighbor pairs (Wilcoxon rank sum *p*-value < 2.2e-16) [Fig. 3D]. Given this feature, we pursued numerous additional analyses of intergenic lncRNA-neighbors and mRNA-neighbors to explore whether positive correlation may be dependent on orientation and/or genomic distance between neighboring loci. In brief, we found that both lncRNAs and mRNAs had a significantly more correlated neighbor (Wilcoxon signed-rank test *p*-value < 2.2e-16 for both lncRNAs and mRNAs), that the distance between intergenic lncRNA-neighbor pairs was not particularly indicative of higher correlation (ρ = −.25), and that lncRNAs and mRNAs were located at comparable distances from other annotated mRNAs (1576 bp versus 1585 bp, respectively) [Additional file 20]. In terms of orientation, we found that expression correlation was equally distributed for tandem (− −> / − −>) and divergent (<− − / − −>) intergenic lncRNA-neighbor pairs, although the expression correlation of convergent (− −> / <− −) pairs was similar to background correlation rates of mRNA-neighbor pairs (Wilcoxon rank sum *p*-value = 0.3607) [Additional file 20]. Taken together, our results indicated that mRNAs, intergenic lncRNAs, and antisense RNAs each have significantly different expression correlation properties with neighboring loci.

We next considered the GC content and length of lncRNA transcripts. The GC content of intergenic lncRNAs was generally lower than that of antisense RNAs, which was lower than that of annotated transcripts [Table 2, Fig. 3E]. This was not surprising given the higher GC content of coding sequences, ribosomal RNA, and transfer RNA in the *P. falciparum* genome [14]. In terms of transcript length, both lncRNA classes were quite long, with the average length of intergenic lncRNAs, antisense RNAs, and annotated transcripts being 1218 bp, 1413 bp, and 2197 bp, respectively [Table 2, Fig. 3F]. The small subset of relatively GC-rich (>29 %) intergenic lncRNAs generally corresponded to the subset of relatively long intergenic lncRNAs (>4 kb), and included all members of the telomere-associated lncRNA-TARE family, whose high GC content and length we previously characterized [arrows in Fig. 3E and F] [41]. The only two unannotated transcripts with greater than 40 % GC content shared 82 % pairwise sequence identity, and they were both situated between *var* pseudogenes and *PHISTB* genes. TransDecoder predicted a coding region in one of these transcripts, and given their high GC content and sequence similarity, we reasoned that both of these transcripts likely represented unannotated pseudogenes.

We further considered the relative location of antisense RNAs within annotated gene bodies and the splicing properties of lncRNAs. This revealed that *P. falciparum* antisense RNAs largely overlapped tail-to-tail with annotated genes, a property that has been described in previous viral, prokaryotic, and lower eukaryotic genome-wide studies [Fig. 3G] [82]. Specifically, the vast majority of *P. falciparum* antisense RNAs initiated transcription downstream of annotated gene bodies and tended to terminate transcription towards the 3’ end of gene bodies as well [Additional file 21]. In terms of splicing, we found that 93.5 % and 89.5 % of predicted intergenic lncRNAs and antisense RNAs were single exon, respectively, versus 47.8 % of annotated transcripts [Table 2].

### Notable LncRNAs

**Fig. 4:**
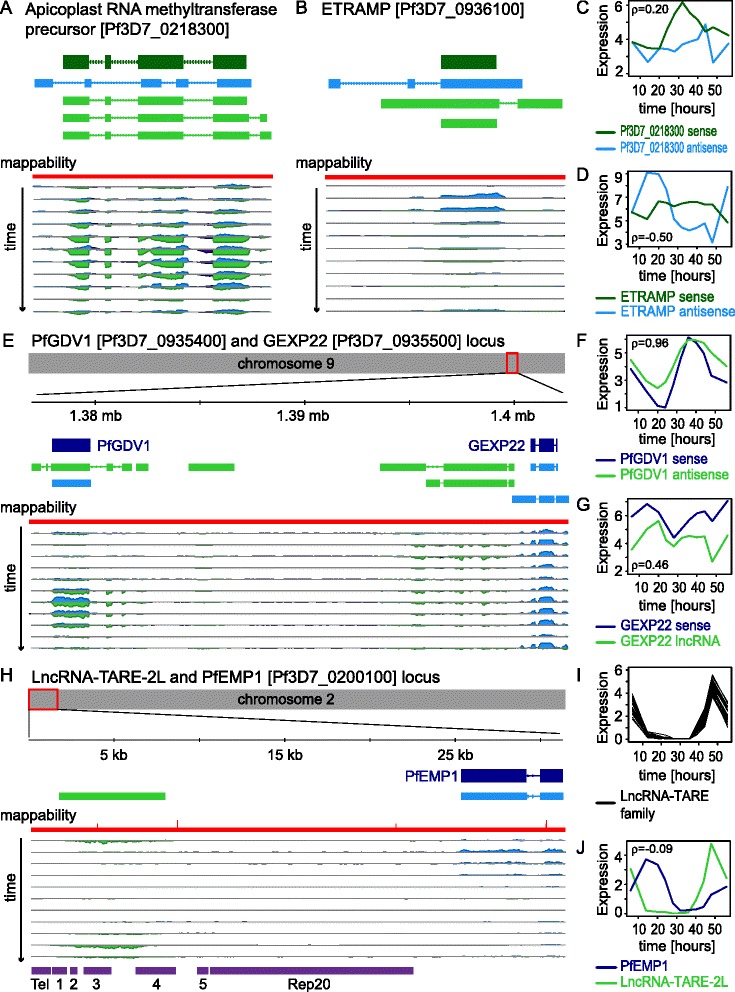
Notable lncRNAs include multi-exonic and telomere-associated transcripts. **(A)**/**(B)** Multi-exonic antisense transcripts span an apicoplast RNA methyltransferase precursor [PlasmoDB:*Pf3D7_0218300*] and an *ETRAMP* [PlasmoDB:*Pf3D7_0936100*] gene, respectively. Annotated gene models are shown in dark green and dark blue, and assembled transcript models are shown in light green and light blue. Reads from each 56-hour time course sample mapping to the (−) strand are shown below each horizontal axis in light green, while reads mapping to the (+) strand are shown above each horizontal axis in light blue. Intron reads are shown in purple. Uniqueness of 100mers is plotted in red as a mappability track. **(C)**/**(D)** Pearson correlation between the *Pf3D7_0218300* sense-antisense pair and *ETRAMP* sense-antisense pair during the 56-hour time course was 0.20 and −0.50, respectively. Notably, *Pf3D7_0218300* and *ETRAMP* antisense transcript levels dropped during parasite invasion, while sense transcript levels did not. Expression is plotted in units of log2(FPKM + 1). **(E)** Multi-exonic lncRNAs are encoded in the *PfGDV1* region on chromosome nine, antisense to *PfGDV1* and between *PfGDV1* and *GEXP22*. Refer to **(A)**/**(B)** for a description of tracks. **(F)**/**(G)** Pearson correlation between the *PfGDV1* sense-antisense pair was 0.96, while Pearson correlation between the divergent intergenic lncRNA and *GEXP22* pair was 0.46 during the 56-hour time course. Expression is plotted in units of log2(FPKM + 1). **(H)** As we have previously described, the telomere-associated repetitive element (TARE) 2–3 region transcribes a family of lncRNA-TAREs, with transcription always proceeding towards the telomere [41]. For example, lncRNA-TARE-2 L is transcribed on the left arm of chromosome two. *Pf3D7_0200100* is a subtelomeric upsB-type PfEMP1-encoding *var* gene. Boundaries of the telomere, TAREs 1–5, and Rep20 are shown in purple. See **(A)**/**(B)** for a further description of tracks. **(I)** Plotting the expression level of 22 lncRNA-TARE family members showed that lncRNA-TARE expression was co-regulated, with maximal firing coinciding with parasite invasion. Expression is plotted in units of log2(FPKM + 1). **(J)** Pearson correlation between lncRNA-TARE-2 L and the neighboring PfEMP1-encoding *var* gene was −0.09 during the 56-hour time course. Expression is plotted in units of log2(FPKM + 1)

Based on the diverse characteristics examined above, we searched for transcripts with exceptional properties. For example, we found that a putative Apicoplast RNA methyltransferase precursor [PlasmoDB:*Pf3D7_0218300*] and an Early Transcribed Membrane Protein [*ETRAMP;* PlasmoDB:*Pf3D7_0936100*] transcribe multi-exonic antisense RNAs across their full gene bodies [Fig. 4A and B]. Expression of the Apicoplast RNA methyltransferase precursor sense-antisense pair was not particularly correlated (ρ = .20), while expression of the *ETRAMP* sense-antisense pair was moderately anti-correlated (ρ = −.50) [Fig. 4C and D]. Interestingly, *ETRAMP* antisense transcription was substantially higher than *ETRAMP* sense transcription, reaching a maximum FPKM of 550 in early stages. This was the highest expression level observed for predicted *P. falciparum* antisense RNAs at any stage. Both the Apicoplast RNA methyltransferase precursor and *ETRAMP* antisense RNAs also demonstrated the 48 hpi expression drop phenomenon, though their sense partners did not exhibit this pattern.

Remarkably, we also found that a region on chromosome nine required for early sexual development [83] harbors a highly expressed, developmentally regulated, five-exon antisense transcript to *P. falciparum* Gametocyte Development Protein 1 [*PfGDV1;* PlasmoDB:*Pf3D7_0935400*], as well as two intergenic lncRNAs downstream of *PfGDV1* [Fig. 4E]. Correlation during the 56-hour blood stage time course between *PfGDV1* sense and antisense transcript levels was the highest of any predicted *P. falciparum* sense-antisense pair (ρ = 0.96), with *PfGDV1* antisense transcript levels typically exceeding *PfGDV1* sense transcript levels [Fig. 4F]. This was in sharp contrast to the majority of *P. falciparum* sense-antisense pairs, which displayed a trend towards anti-correlated expression. Notably, while the expression correlation was again high between *PfGDV1* sense-antisense transcript levels in the four biological replicate samples (ρ = 0.85), the difference between transcript levels was greater, with the *PfGDV1* antisense transcript reaching a maximum FPKM of 255 [Additional file 22]. The nearby, multi-exonic, intergenic lncRNA exhibited moderately correlated expression to GEXP22 [PlasmoDB:*Pf3D7_0935500*] and evidence of alternative splicing (ρ = 0.46) [Fig. 4G]. In summary, the *PfGDV1* antisense transcript’s expression properties, multi-exonic structure, and position relative to other genes made it a clear outlier in the genome.

While we have previously detected and characterized telomere-associated lncRNA-TAREs, the properties of this yet to be annotated lncRNA family again stood out in our analyses [41]. Our results confirmed that lncRNA-TAREs were long, high-GC, and transcribed towards the telomere [Arrows in Fig. 3E and F, Fig. 4H]. We also confirmed that lncRNA-TARE transcription was generally restricted to the expected TARE 2–3 region, although we did find that in one case the entire TARE 1–6 region was transcribed [Additional file 23]. To build on our previous results, long, paired-end, uniquely mapped sequencing reads showed that lncRNA-TARE transcripts likely originated from 22 chromosome ends in our parasite populations. Moreover, the increased time resolution and scope of our samples showed that lncRNA-TARE transcript levels coordinately peaked during parasite invasion [Fig. 4I]. Interestingly, we found that sterile *var* transcript levels peaked during parasite invasion as well, but that not all *var* genes produced these non-coding transcripts [Additional file 24] [84]. For example, the subtelomeric *var* gene [PlasmoDB:*Pf3D7_0200100*] neighboring lncRNA-TARE-2L was lowly expressed during the ring stage and did not produce appreciable sterile transcripts [Fig. 4H and I]. Collectively, these findings suggested co-regulated firing and coordinated function of lncRNA-TARE and sterile *var* transcripts during parasite invasion.

### LncRNA structural validation

To facilitate the future study of lncRNAs, we sought to experimentally confirm novel lncRNA transcript structures using PCR and Sanger Sequencing. Towards this end, we amplified and sequenced across splice junctions predicted within the five-exon Apicoplast RNA methyltransferase precursor antisense transcript, three-exon *ETRAMP* antisense transcript, and five-exon *PfGDV1* antisense transcript. In total, Sanger sequencing results confirmed nine lncRNA junctions [Additional files 25 and 26].

### Discovery and validation of CircRNAs in P. falciparum

To globally investigate RNA circularization in *P. falciparum,* we used the analysis pipeline and criterion published by Memczak et al. [55]. This approach identified 1381 putative *P. falciparum* circRNAs with at least two unique reads spanning their splice junction (between 0.1 and 10 kb long) [Fig. 1A, Additional file 27] [55, 85]. Of these, 273 had five or more unique reads of support (the gold standard being 2 reads). As compared to the transcriptome-wide results reported in Table 2, we found that *P. falciparum* transcripts with predicted circRNAs were more highly expressed on average (set metrics: average of maximum FPKMs 2646; average of average FPKMs 791). Indeed, the circRNA-producing gene set was enriched for ribosome-related components; ribosomal proteins are typically highly expressed [Additional file 28].

In contrast to human circRNAs, *P. falciparum* circRNAs were generally predicted to be short, with the majority being less than 200 bp [56]. Only 509 out of 1381 predicted circRNAs with at least two unique supporting reads were predicted to be 200 bp or longer. In the more stringent set of 273 circRNA candidates with at least five unique supporting reads, only 72 were predicted to be 200 bp or longer. We defined circRNA size as the genomic distance between predicted donor site and acceptor site, inclusive of the donor and acceptor site. Thus, this should be read as a maximum size, as circRNAs can span introns, which may be spliced out of the circRNA sequence. In summary, short circRNAs appeared to outnumber longer circRNAs in *P. falciparum* and deserve further attention*.*

**Fig. 5:**
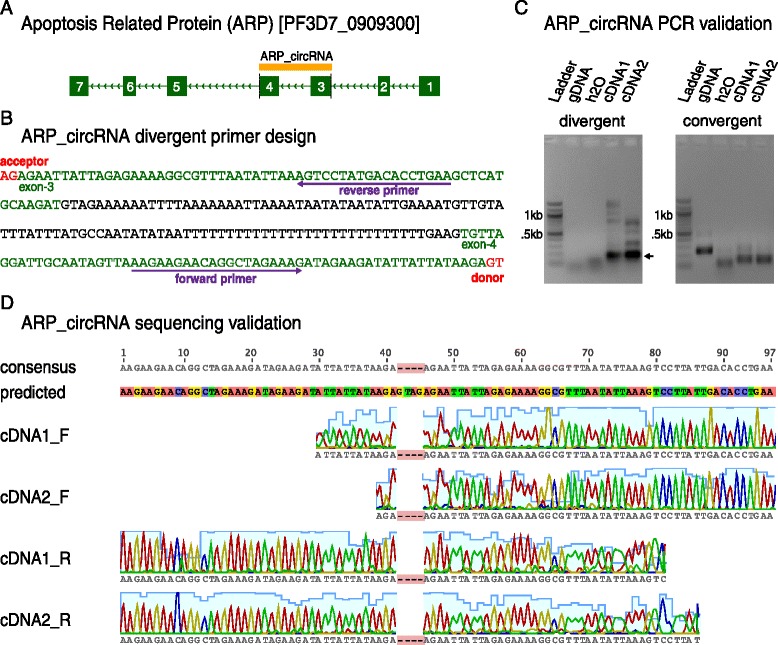
Divergent primers and Sanger sequencing validate circRNA splicing in *P. falciparum.* **(A)** The apoptosis-related protein (*ARP*) encodes a predicted circRNA, termed ARP_circRNA, consisting of *ARP* exon-3 and exon-4 sequence. **(B)** To validate the non-canonical exon-4 donor (GT)/exon-3 acceptor (AG) splice junction in ARP_circRNA, we designed a divergent PCR primer pair. The primer pair is considered to be divergent, rather than convergent, because the reverse primer binds upstream of the forward primer. **(C)** PCR using divergent primers amplified a product of the expected size (161 bp, indicated with an arrow) when the template was cDNA from either time course, but not water or gDNA. The larger products in the divergent cDNA reactions may represent non-specific or rolling-circle reverse transcription products [45]. On the other hand, PCR using convergent primers amplified products of the expected size when the template was cDNA from either time course or gDNA. We confirmed that the smaller product size in the case of convergent cDNA reactions corresponded to intron removal. **(D)** Sanger sequencing of divergent amplicons of the expected size confirmed the ARP_circRNA junction in both time courses. The extra GTAG in the predicted sequence marks the non-canonical ARP_circRNA splice junction (highlighted in red in the consensus sequence)

We predicted an intriguing top *P. falciparum* circRNA candidate within the apoptosis-related protein [*ARP;* PlasmoDB:*Pf3D7_0909300*], termed ARP_circRNA [Fig. 5A]. 56 unique reads spanned the predicted splice junction between ARP’s exon-4 donor site (GT) and upstream exon-3 acceptor site (AG) [Fig. 5B]. To validate that this non-canonical splice junction was not the result of a library preparation or sequencing artifact, we reverse-transcribed total RNA and amplified the predicted ARP_circRNA junction from the resulting complementary DNA (cDNA) using PCR and divergent primer pairs. We designed divergent ARP_circRNA primer pairs, as is depicted in Fig. 5B, such that primer pairs could not amplify genomic DNA (gDNA) or cDNA in the absence of the predicted ARP_circRNA splice junction.

Our results confirmed the non-canonical ARP_circRNA splice junction in cDNA preparations from either biological replicate time course. Specifically, the ARP_circRNA divergent primer pair produced amplicons of the expected size when the template was cDNA, and did not produce specific amplicons with gDNA or water as the template. On the other hand, the ARP_circRNA convergent primer pair amplified both cDNA and gDNA, with the smaller product size in the cDNA reactions corresponding to intron removal [Fig. 5C]. We further confirmed the identity of the ARP_circRNA divergent and convergent amplicons by Sanger sequencing. Sequence confirmation for the ARP_circRNA divergent amplicon is shown in Fig. 5D, where the GTAG splice donor-acceptor tag is included in the predicted sequence as a marker for the circRNA splice junction.

We used the same experimental strategy of divergent PCR followed by Sanger sequencing to validate additional *P. falciparum* circRNA candidates. In total, we were able to validate six out of nine tested candidates [Additional files 29 and 30]. We selected the nine tested candidates according to certain criterion: read support, a donor or acceptor site in common with an annotated transcript, predicted size of at least 200 bp (genomic distance), and not within a ribosomal gene. Two of the additional validated *P. falciparum* circRNAs were associated with genes of unknown function, two were predicted within rhoptry-related genes, and the final validated candidate was within metacaspase-like protein (MCA2), which is another gene involved in apoptosis. As has been suggested across other organisms, temporal expression of validated circRNAs was moderately correlated with that of their linear counterparts [Column Q in Additional file 27] [86, 87].

Interestingly, using the recently described PACCMIT-cds algorithm, we found that our experimentally validated circRNA candidates each contained predicted human microRNA binding sites [Additional file 31] [88]. Moreover, when we broadly searched *PlasmoDBv10.0* transcripts for human microRNA binding sites, we found thousands of significant hits and that 61 transcripts harbored at least 100 predicted binding sites for a given human microRNA (*p*-value < 0.05) [Additional file 32]. At the highest stringency level (*p*-value < 1.0e-6), the gametocyte-specific transcript Pf11-1 [PlasmoDB:*Pf3D7_1038400*] harbored an impressive 1569 predicted human microRNA binding sites. Taken together, we have predicted an unexpectedly widespread capacity for *P. falciparum* transcripts to form stable circular structures, as well as to bind human microRNAs.

## Discussion

The mechanisms underpinning gene regulation in *P. falciparum* malaria remain largely uncharacterized [34, 35]. However, long non-coding RNAs (lncRNAs) have been found to initiate and guide the transcriptional, post-transcriptional, and epigenetic status of specific loci across a broad range of organisms [47, 48]. Encouraged by these features and our previous discovery of an intriguing family of telomere-associated lncRNAs in *P. falciparum*, we have developed strand-specific *P. falciparum* RNA sequencing methods, deeply sequenced fifteen blood stage samples, and compiled a comprehensive catalog of *P. falciparum* lncRNA transcript properties.

Our results have several implications for parasite biology. For example, we observed numerous negatively correlated, tail-to-tail overlapping sense-antisense transcript pairs. This is consistent with a potential role for many *P. falciparum* antisense RNAs in transcriptional and/or post-transcriptional regulation of their sense mRNA partners [48]. For example, a subset of *P. falciparum* antisense RNAs may function through transcriptional interference, as has been extensively studied in *Saccharomyces cerevisiae* [82, 89–91]*.* In the transcriptional interference model, antisense transcription interferes with sense transcription through either polymerase collisions or alternative mechanisms. As an alternative or additional model, antisense-mediated transcriptional suppression is also possible and has been described in human studies [92–94]. In antisense-mediated transcriptional suppression, antisense RNAs act as epigenetic silencers, catalyzing local heterochromatin formation.

We also observed rapid depletion of antisense transcript levels (and some mRNA transcript levels) during invasion. This pattern is intriguing and suggests that a specific subset of transcripts may be targeted for degradation during this critical timeframe. Notably, we were not able to identify evidence of degraded transcripts in our dataset, though the size selections imposed during library preparation would likely eliminate such fragments.

Searching our catalog for *P. falciparum* lncRNAs with unique properties revealed that an essential protein in early gametocyte development, *PfGDV1,* has a highly and coordinately expressed, multi-exonic antisense counterpart*,* as well as multiple neighboring intergenic lncRNAs. Though the regulation and mechanism of early gametocyte development events remain largely unknown, Eksi et al. have shown that *PfGDV1* complementation restores gametocytogenesis in *PfGDV1-*null parasites, and that episomal *PfGDV1* over-expression increases gametocytemia in wild-type parasites [83]. This suggests that endogenous *PfGDV1* expression levels likely correlate with gametocytemia, and that silencing the endogenous *PfGDV1* locus could disrupt transmission. Notably, Kafsack et al. have also shown that a member of the ApiAP2 transcription factor family is involved in early gametocyte development [36]. However, loss of this factor did not affect *PfGDV1* transcript levels [36], suggesting that the *PfGDV1* locus may integrate different or additional regulatory signals.

In light of these recent findings, and given that future strategies to block malaria transmission largely hinge on blocking the development of transmissible *P. falciparum* sexual stages, we highlight here the need for further study of the *PfGDV1*-associated lncRNAs [11–13]. Specifically, we propose that single-cell experiments and dissection of the *PfGDV1* locus using genome editing techniques may reveal a regulatory role for *P. falciparum* lncRNAs in early gametocyte commitment events, perhaps similar to *S. cerevisiae* lncRNA-mediated entry into meiosis [51, 52]. In this system, lncRNA transcription through the *S. cerevisiae* Inducer of Meiosis *IME1* promoter region and *IME4* antisense transcription are incompatible with *IME1/IME4* sense transcription.

We have previously described and hypothesized that a family of telomere-associated lncRNA-TARE transcripts is involved in telomere maintenance and/or subtelomeric *var* gene regulation [41, 95]. Supporting such nuclear roles, Siegel et al. then reported significant enrichment of telomere-associated *P. falciparum* lncRNAs in the nuclear, as opposed to total and cytoplasmic, *P. falciparum* RNA fractions [44]. Adding to the lncRNA-TARE family profile, we have now shown that lncRNA-TARE transcripts are maximally expressed during parasite invasion, along with the sterile, non-coding *var* transcripts transcribed from bidirectional *var* intron promoters [84]. When parasites invade RBCs pre-loaded with episomal PfEMP1-encoding *var* genes, these episomal *var* genes are not silenced during the first ring stage. *Var* gene silencing is, however, somehow established by the subsequent ring stage and then remains the default status [96]. While it has been hypothesized that passage through S-phase is the critical requirement for *var* gene silencing, we suggest that expression of non-coding transcripts during parasite invasion may play an important regulatory role as well [97, 98]. High-resolution studies of chromatin mark dynamics over episomal and endogenous *var* loci during parasite invasion may help to resolve these models.

This work represents the validation of six *P. falciparum* circRNAs, as well as the prediction of hundreds more. We stress, however, that further curation of our circRNA predictions will be required, as *in silico* prediction algorithms may yield false positives and PCR failures may yield false negatives for low copy number targets or otherwise. With this said, a more focused sequencing effort using circRNA-specific protocol adjustments may reveal even more abundance of this intriguing class of non-coding RNA. In support of this notion, most of our predicted *P. falciparum* circRNAs were shorter than the average library fragment size, and thus could be selected against during library preparation. Moreover, Wang et al. recently mined the Siegel et al. RNA sequencing data set and validated two low-abundance *P. falciparum* circRNAs [44, 45]. As we did not find evidence for either of these candidates in our study, this further hints that library preparation and/or bioinformatic processing may be tunable to capture additional *P. falciparum* circRNAs. Alternatively, as our sample sets differed, these circRNAs may be highly linked to the phenotypic state of the parasite.

Notably, Memczak et al. and others have hypothesized that circRNAs may function as microRNA sponges, and have demonstrated a handful of supporting examples [55, 56]. In this model, the circRNAs behave as competitive inhibitors, sponging up microRNAs to reduce the microRNA pool available to bind messenger RNA targets. Given that the lack of *P. falciparum*-encoded microRNAs and microRNA processing machinery has been extensively established, we did not anticipate microRNA sponge roles for *P. falciparum* circRNAs [39, 40, 58]. Moreover, the only reported function for human microRNAs in *P. falciparum* involves microRNA integration into parasite transcripts rather than binding interactions [40, 57]. Nonetheless, we were curious to investigate the capacity for human microRNAs to bind within our set of validated circRNA transcripts and within *P. falciparum* transcripts more generally. Challenging our *a priori* expectations, we predicted human microRNA binding sites within each validated circRNA, as well as across thousands of *P. falciparum* transcripts. Taken together, our results may point to additional functions for circRNAs across eukaryotes and/or to undiscovered functions for human microRNAs in *P. falciparum* gene regulation*.*

## Conclusions

In this work we develop strand-specific non-polyA-selected next generation sequencing methods sensitive to the extreme AT content of *P. falciparum.* We then apply these methods to the transcriptome-wide assembly and characterization of *P. falciparum* intergenic lncRNA and antisense RNA properties. Our results support the conserved regulatory capacity of non-coding elements in *P. falciparum*, with different transcripts demonstrating distinct regulatory signatures, such as stage-specific expression, rapid firing, rapid destabilization, transcriptional interference, and circularization. In addition to a highly curated lncRNA transcript catalog, we provide structural validation of three exceptional multi-exonic lncRNAs and six circRNAs. This work coupled with recent advancements in *P. falciparum* genome editing will greatly facilitate further insights into the function of these lncRNAs in *P. falciparum* [99, 100].

## Methods

### Ethics statement

The Harvard University Human Subjects Committee deemed this research to be exempt from continuing review (Application Number: F19615-102), as samples were sourced from publicly available, preexisting, de-identified repositories.

### Parasite culture and sample harvesting

We harvested a total of fifteen samples from two independent *P. falciparum* blood-stage time courses. In each experiment, we cultured a freshly thawed *P. falciparum* strain 3D7 clone (ATTC) in human RBCs from healthy anonymous donors (Research Blood Components, Boston, MA) using standard methods [10]. We maintained cultures at 4 % hematocrit and supplemented RPMI-HEPES medium with 5 % human serum (O+) and 5 % Albumax II (Gibco). We synchronized cultures using multiple 5 % sorbitol solution treatments [101], and expanded cultures to accommodate harvesting of at least 50 mL of culture at each planned time-point (time course 1: every ~4 h for 56 h; time course 2: ~4 h before and after the ring to trophozoite and trophozoite to schizont morphological stage transitions). We centrifuged 50 mL aliquots of harvested culture at 2400 rpm in a Sorval RT6000B to obtain at least 2 mL of packed RBCs per time-point. For time course 1, we stored packed RBCs in 15 mL of Buffer RLT (with BME added) at −80 °C prior to RNA extraction. For time course 2, we lysed packed RBCs using a .05 % saponin solution, washed liberated parasites using phosphate-buffered saline (pH 7.4), pelleted parasites at 13.2 RPM in a micro-centrifuge, and stored parasites in 1 mL of TRIZOL reagent at −80 °C prior to RNA extraction.

### Total RNA extraction

For time course 1, we thawed RBC samples stored in Buffer RLT, added one volume of 70 % ethanol, and immediately loaded the mixture onto RNeasy Midi columns (Qiagen). For time course 2, we thawed parasite samples stored in TRIZOL reagent, performed TRIZOL-chloroform extraction, and immediately applied the aqueous layer to RNeasy Mini columns (Qiagen). During both RNeasy Midi and Mini RNA extraction procedures, we performed the optional on-column DNase I digestion for thirty minutes to remove genomic DNA. We stored isolated total RNA aliquots at −80 °C with 1 unit/uL RNaseOUT (Invitrogen), and validated RNA quality using an Agilent Bioanalyzer RNA 6000 Pico Kit.

### Strand-specific, Non-polyA-selected, library preparation and sequencing

We began library preparation with a second DNase treatment (Ambion TURBO DNase) using 20 units of SUPERase-In (Ambion) and 40 units of RNaseOUT (Invitrogen) to protect RNA from degradation. Each DNase reaction was incubated at 25 °C for 30 min followed by 1.8X RNAclean SPRI bead purification (Agencourt). Second, we used a Human/Mouse/Rat Ribo-Zero Magnetic Kit (Epicentre) to deplete 18S and 28S rRNA from DNase-treated total RNA. We used 3.5-5 ug of DNase-treated total RNA for all samples except T6, T14, and TT8. In these cases, we used .4 ug, 1 ug, and .7 ug of DNase-treated total RNA, respectively. Furthermore, for T6 we mixed 4 and 8 hpi total RNA 1:1, and for T14 we mixed 12 and 16 hpi total RNA 1:1. Third, we fragmented rRNA-depleted RNA at 85 °C for 8 min using Mg^2+^ Fragmentation Buffer (New England Biolabs), followed by 1.8X RNAclean SPRI bead purification (Agencourt). Fourth, we reverse-transcribed the fragmented RNA using SuperScript III (Invitrogen), 200 ng/uL of freshly prepared Actinomycin D (Sigma-Aldrich), 3 ug of 76 % AT-biased random hexamers (Integrated DNA Technologies), and a gradually ramping-up thermocycler program. Specifically, we set the ramp speed of a PTC-225 DNA Engine Tetrad (MJ Research) to .1 °C/sec and used the following program: 5 °C, 10 °C, 15 °C, 20 °C, 25 °C, 30 °C, 35 °C for 5 min each, 42 °C for 30 min, 45 °C, 50 °C, 55 °C for 10 min each. We cleaned up first strand synthesis (FSS) reactions using both Micro Bio-Spin P-30 RNase-free columns (Bio-Rad) and 1.8X RNAclean SPRI bead purification (Agencourt). Fourth, we performed second strand synthesis (SSS) using a biased dACG-TP/dU-TP mix (Fermentas), 10 units of *E. coli* DNA ligase (Invitrogen), 160 units of *E. coli* DNA polymerase (Invitrogen), and 2 units of *E. coli* RNase H (Invitrogen), followed by 1.8X AmpureXP SPRI bead purification (Agencourt). Fifth, we used an Illumina series KAPA Library Preparation Kit (Kapa Biosystems) and barcoded Y-adapters developed by the Broad Institute [Additional file 25] to end repair, A-tail, and ligate adapters to each library. We added adapters in approximately 15-fold excess of library targets, and removed un-ligated adapters and adapter-dimers using 1.0X AmpureXP SPRI bead purification (Agencourt). Sixth, we digested the dUTP-marked second strand at 37 °C for 30 min, followed by 25 °C for 15 min, using Uracil-Specific Excision Reagent (USER) enzyme (New England Biolabs). Seventh, we amplified libraries for as few cycles as necessary using a KAPA Real-Time PCR Library Amplification Kit (Kapa Biosystems) and PCR primers developed by the Broad Institute [Additional file 25]. Each library except for T6, T14, and TT8 required only four PCR cycles, while T14 and TT8 required eight PCR cycles, and T6 required twelve PCR cycles. Following a 2 min denaturation step at 98 °C, we cycled libraries using an ABI 7900 Real-Time PCR machine and the following 2-step program: (1) denaturation at 98 °C for 20 sec, (2) annealing and extension at 55 °C for 190 sec.

Finally, we quantified libraries using a KAPA Library Quantification Kit (Kapa Biosystems) and an Agilent Bioanalyzer High Sensitivity DNA Kit. We combined barcoded libraries into two pools, and sequenced each pool on an Illumina Hiseq 2000 machine (one lane per pool) using 101-bp, paired-end read technology. We prepared the fifteen libraries used in this study in parallel (except for real-time amplification). We used the FASTX-Toolkit v0.0.13 to assess raw read quality. See Additional files 1 and 2 for a library preparation flowchart and a detailed library preparation protocol, respectively.

### Read processing, TopHat read alignment, and aligned read benchmarking

We trimmed the last base from reads passing Illumina filtering (PF) using Picard release 1.94 (SamToFastq). We then aligned reads from each sample to the *PlasmoDBv10.0* reference genome using TopHat v2.0.9 and the following parameters: −r 300 –mate-std-dev 100 –library-type fr-firststrand –i 70 –I 5000 –read-mismatches 0 –segment-mismatches 0 –max-segment-intron 5000 –max-coverage-intron 5000 –b2-very-sensitive –read-gap-length 0 –read-edit-dist 0 –read-realign-edit-dist 0 –max-deletion-length 0 –max-insertion-length 0 –max-multihits 2 –no-mixed –no-discordant [66]. These parameters specified an average fragment size of 300 bp with a standard deviation of 100 bp, strand-specific reads prepared using the dUTP method, and an expected intron size of 70–5,000 bp. Also, to allow only perfect read pair mapping in the expected orientation, and to report only two alignments for non-unique read pairs.

To remove non-unique reads, we then used SAMtools v0.1.19 (view –q 10) to remove read pairs with a mapping quality of 10 or less, meaning at least a 10 % chance that the read pair truly came from somewhere else in the genome. We used SAMtools v0.1.19 (flagstat) to compile the unique read alignment rate per sample. Subsequently, we used RNA-SeQC hosted by Genepattern to calculate the strand-specificity, exonic rate, intronic rate, intergenic rate, gap rate, and coefficient of variation of the top 1000, 2000, and 4000 expressed genes for each set of aligned reads [67, 102]. We also used RSeQC to calculate the rRNA rate and insert size of aligned reads, and the GC content of rRNA-filtered, aligned reads [68]. We used the FASTX-Toolkit v0.0.13 to assess aligned read quality.

We generated the mappability track displayed (in red) in the Array Studio version 7.0 Genome Browser (OmicSoft Corporation) screen shots using the following procedure: (1) We shredded the *PlasmoDBv10.0* genome (excluding mitochondrial and apicoplast contributions) into every possible 100mer sequence. (2) We used the R-project Biostrings package to search for each 100mer sequence in the *PlasmoDBv10.0* genome. (3) We scored the mappability of each 100mer by tallying the number of exact matches possible. (4) Lastly, we viewed the minimum mappability score of 100mers along the genome, such that deviations above the baseline (score of one) indicated regions where 100-bp *single-end* reads could not uniquely align [Additional file 33]. We calculated that 96.53 % of all possible 100mers in the *PlasmoDBv10.0* genome are unique. We used MUSCLE to compute the pairwise sequence identity between *CLAG3.1* and *CLAG3.2* coding regions (96.4 %) [103].

### Cufflinks assembly

We used Cufflinks v2.1.1 to assemble aligned reads from each sample into transfrags using the following parameters: −library-type fr-firststrand –max-intron-length 5000 –overlap-radius 1 –min-isoform-fraction .25 –pre-mrna-fraction .25 –min-frags-per-transfrag 50 –trim-3-dropoff-frac .2 –frag-bias-correct [76]. These parameters specified strand-specific reads prepared using the dUTP method and a maximum intron size of 5,000 bp. Also, to not merge transfrags, to filter minor isoforms less than 25 % as abundant as the major isoform, to ignore intronic alignments if not as abundant as specified, to require at least 50 aligned read fragments per assembled transfrag, to trim the 3’ end of assembled transfrags at 20 % of average coverage, and to run the built-in bias correction algorithm prior to estimating transcript abundance. We also specified a set of 374 short (<300 bp) and/or structural RNAs (all *PlasmoDBv9.3* transcripts with class code: tRNA, rRNA, or snoRNA) to exclude from assembly (and abundance estimation), such that analyses relying on expression profiles correspond to mRNAs [Additional file 34]. We used Cuffmerge to parsimoniously merge assembled transfrags from each sample into a final transcriptome assembly, with or without incorporating *PlasmoDBv10.0* annotated transcript models (RABT) [74].

### Genome-guided trinity assembly

We merged all trimmed reads passing Illumina filtering (PF), aligned merged reads to the *PlasmoDBv10.0* genome using GSNAP release 2013-11-27, and then used scripts from Trinity release 2013-11-10 to assemble a genome-guided *P.falciparum* transcriptome (PASA release 2013-09-07, GMAP release 2013-11-27, Blat v34) [75]. We specified a maximum intron size of 5,000 bp and strand-specific reads prepared using the dUTP method where appropriate. We also parsimoniously merged the genome-guided Trinity assembly with *PlasmoDBv10.0* annotated transcript models (using Cuffmerge [74]) to yield a Genome-guided Trinity RABT assembly.

### Assembly performance assessment

To assess Cufflinks and genome-guided Trinity assembly performance, we used Cuffcompare to calculate the total number of assembled transcripts, the number of intergenic transcripts, and the number of antisense transcripts [74]. We also transformed assembly GTF files into BED12 files, and used BEDTools v2.18.2 to investigate assembly contiguity and chimerism [104]. We defined contiguity as the rate of *PlasmoDBv10.0* annotated transcripts covered by one assembled transcript over at least 90 % of the annotated transcript exonic length in the correct orientation. We defined chimerism as the rate of assembled transcripts that span more than one *PlasmoDBv10.0* annotated locus in the correct orientation. For contiguity rate, we used the following BEDTools command: < bedtools intersect –split –s –f .90 –wa –wb –a *PlasmoDBv10.0.*bed12 –b Assembly.bed12 >. For chimerism we used the following BEDTools command: < bedtools intersect –split –c –s –a Assembly.bed12 –b *PlasmoDBv10.0*.bed12 >, followed by brief post-processing.

We next paired 5727 and 7736 assembled transcripts from the Cufflinks and Cufflinks RABT transcriptomes, respectively, with *PlasmoDBv10.0* annotated transcripts, and computed the Pearson correlation between paired expression profiles. Previously unannotated assembled transcripts (including antisense) were naturally excluded from pairings. We calculated a median correlation of .98 and .99 for Cufflinks and Cufflinks RABT paired expression profiles, respectively. We also compared the following properties of paired Cufflinks transcripts, paired Cufflinks RABT transcripts, and *PlasmoDBv10.0* annotated transcripts: average of maximum FPKMs, average of average FPKMs, average length, average GC content, single exon rate, and maximum exon count. We only considered exonic sequence in transcript length and GC content calculations [Additional files 12 and 13].

### Coding region prediction

We used TransDecoder release 2013-11-17 to predict coding regions in the Cufflinks RABT transcriptome [75]. TransDecoder identifies regions in spliced transcript models that likely encode peptides greater than 100 amino acids based on (1) a match to a Pfam domain above the noise threshold or (2) a log-likelihood score of a Markov model for coding DNA that is greater than zero and greatest when calculated in the predicted open reading frame. Using this approach, we predicted coding regions in 5213 out of 5229 assembled protein-coding transcripts at least 100 amino acids long (99.7 %), versus just 7 out of 660 intergenic transcripts (1.1 %) and 11 out of 474 antisense transcripts (2.3 %).

To estimate the background rate of coding potential detected by TransDecoder, we used BEDTools v2.18.2 (shuffle) to randomly generate 6600 size-matched intergenic intervals and 4740 size-matched antisense intervals [75, 104]. Following minor post-processing, we paired the shuffled regions with *PlasmoDBv10.0* transcript models (to train TransDecoder) and assessed coding potential prediction. TransDecoder predicted coding regions in 97 out of the 6600 random intergenic regions (1.5 %) and 57 out of the 4740 random antisense regions (1.2 %). We concluded from this analysis that the rate of coding potential in the lncRNA transcript sets was not significantly different from the background rate in size-matched shuffled regions (Fisher’s exact test, *p*-value = .493 for intergenic regions; Fisher’s exact test, *p*-value = .053 for antisense regions).

### Differential expression and regulation

To assess differential expression and regulation of both the *PlasmoDBv10.0* and the Cufflinks RABT transcriptomes, we used Cuffdiff v2.1.1 and the following parameters: −library-type fr-firststrand –time-series –min-reps-for-js-test 1. These parameters specified strand-specific reads prepared using the dUTP method, time course samples, and to not require replication of all conditions. We again specified a set of 374 short (<300 bp) and/or structural RNAs (all *PlasmoDBv9.3* transcripts with class code: tRNA, rRNA, or snoRNA) to exclude from abundance estimation, such that analyses relying on expression profiles correspond to mRNAs [Dataset 32].

We instructed Cuffdiff to compare samples from the 56-hour time course harvested approximately 8 h apart (T6 vs. T14, T14 vs. T24, T20 vs. T28, T24 vs. T32, T28 vs. T36, T32 vs. T40, T36 vs. T44, T40 vs. T48, T48 vs. TT8) and to estimate biological variation across the blood stage using the four replicated conditions [74]. Specifically, we considered the correlation matrix between sample profiles and paired R with T14 and T20, ET with T28, LT with T32, and S with T40. We then supplied these replicated conditions to Cuffdiff as samples in addition to specifying the desired sample comparisons described above. Finally, we used the Benjamini and Hochberg method and a FDR cut-off of 5 % to threshold significance [105].

After removing chimeric results, our analysis of the Cufflinks RABT transcriptome predicted 3815 differentially expressed genes, 127 alternative splicing events, and 81 cases of alternative promoter usage [Additional files 35, 36 and 37]. This included 354 significant intergenic lncRNA loci (out of 647) and 69 significant antisense loci (out of 467). We noted an absence of strong correlation between maximum sense and antisense partner expression [ρ = .19, Additional file 38], which reiterated the strand-specificity of our sequencing reads and the integrity of the antisense signal. Analyzing the *PlasmoDBv10.0* annotated transcriptome alone predicted 4284 differentially expressed mRNA genes.

### GO term enrichment

We defined 1632 ring-specific genes as differentially expressed, annotated genes with maximum expression between 6–20 hpi during the 56-hour time course. Similarly, we defined 1378 trophozoite-specific genes (24–36 hpi maximum expression) and 1274 schizont-specific genes (40–48 hpi maximum expression). We converted *PlasmoDBv10.0* gene annotations to SangerDB *P. falciparum* gene annotations, and then used GOstat and a FDR cut-off of 1 % to search for biological process GO terms significantly over-represented in the ring-specific, trophozoite-specific, and schizont-specific gene sets [73]. See Additional file 7 for stage-specific gene sets and results.

We intersected the 1381 predicted circRNA loci with *PlasmoDBv10.0* gene annotations to yield a circRNA-producing gene set, and used an analogous approach to search for significantly over-represented GO terms. In the circRNA GO term analysis, we included biological process, molecular function, and cellular component results [Additional file 28].

### Sample staging and expression profile visualizations

To visualize sample similarities on a transcriptome-wide scale, we first constructed a distance matrix of sample expression profiles as follows: (1) we only considered contributions from the 5077 annotated mRNAs with an FPKM rising above one during the 56-hour time course, (2) we transformed FPKM values as log2(FPKM + 1), (3) we computed the Pearson correlation (ρ) matrix between sample expression profiles, and (4) we transformed this matrix as (1- ρ)/2. We then used MDS as implemented by the R-project *cmdscale* function to visualize sample expression profile similarities in two dimensions.

To visualize differentially expressed, annotated mRNA expression profile dynamics during the 56-hour time course, we followed steps 1–2 as above using the 4284 differentially expressed mRNA genes, and then used the *circularmap* function distributed in the R-project NeatMap package [Additional file 39] [71]. The NeatMap package implements the non-metric MDS algorithm previously proposed by Taguchi and Oono [71, 106]. We used the more traditional *make.heatmap1* function distributed in the R-project NeatMap package to compare the expression profiles of lncRNAs and annotated mRNAs (FPKM >0) during the 56-hour time course.

### Upstream motif prediction

To search for regulatory motifs amongst co-expressed transcripts, we extracted the 1 kb of (+) strand sequence upstream from both assembled mRNA and lncRNA transcripts (excluding chimeras). We then searched both strands using a standalone installation of the RED^2^ motif prediction algorithm [79]. We selected RED^2^, as this clustering-free approach has both recently been applied to *P. falciparum* and shown to detect motifs that clustering based approaches did not. We specified the mutual information scoring function, a maximum motif size of 15, to perform profile normalization, and to use 100 random replicates in estimating the global FDR. This analysis returned 66 significant motifs (FDR 0.001), many shared between mRNAs and lncRNAs. Our results are included in Additional file 19.

### Stage specificity score

To summarize transcript stage specificity in a single quantitative metric, we calculated a maximum transcript specificity score that is based on the Jensen-Shannon (JS) divergence. This metric has been fully described by Cabili et al. and was used by this group to annotate the tissue specificity of human intergenic lncRNA transcripts [107]. Specifically, we calculated specificity scores for each transcript, inspected the top 5 % most stage-specific transcripts, and compared transcript specificity scores by transcript class. We normalized transcript expression levels as in Cabili et al. and used the *shannon.entropy, JSdistVec,* and *JSdistFromP* functions implemented in the R-project CummeRbund package, except that we used a logarithmic base of two in the Shannon entropy calculation to match the Cabili et al. methods [74, 107]. We also performed this analysis on the set of 1619 annotated mRNAs with an average FPKM of less than 20 (set metrics: average of average FPKMs 1.95; average of maximum FPKMs 4.3).

Corroborating our visual observations, each telomere-associated lncRNA-TARE transcript was in the set of top stage-specific transcripts [column R in Additional files 15 and 16]. However, global comparisons of lncRNA specificity scores and mRNA specificity scores were less conclusive; lncRNAs appeared to be more stage-specific than the full set of annotated mRNAs, but this difference was not clear when we compared lncRNAs to only lowly expressed mRNAs [Additional file 40]. Thus, while the specificity metric accurately summarized the increased stage specificity of certain transcripts, such as the telomere-associated lncRNA-TAREs, further comparisons will be necessary to make any global conclusions as to the increased or equivalent specificity of *P. falciparum* lncRNAs versus mRNAs.

### Expression correlation

To generate a null distribution of location-independent gene pair correlation, we investigated the Pearson correlation during the 56-hour time course between 50,000 random *PlasmoDBv10.0* mRNA gene pairs. We then computed the Pearson correlation between 5251 mRNA-neighboring gene pairs, 498 intergenic lncRNA-neighboring gene pairs, and 445 sense-antisense gene pairs, and compared these distributions to the null distribution of location-independent gene pair correlation. To be consistent, we defined the neighboring gene of both mRNAs and intergenic lncRNAs as their more correlated neighboring mRNA (unless otherwise specified). We excluded gene pairs from analysis when one of the genes was a short and/or structural RNA [Additional file 34], or otherwise had a FPKM of zero during the 56-hour time course. Also, we excluded the result if the more or less correlated neighbor of an intergenic lncRNA was another intergenic lncRNA.

Using this approach, we found a significant shift towards positive correlation in the case of both mRNA-neighboring genes and intergenic lncRNA-neighboring genes (Wilcoxon rank sum *p*-value < 2.2e-16 in both cases), and a significant shift towards negative correlation in the case of sense-antisense genes (Wilcoxon rank sum *p*-value = 3.834e-11). Our results also indicated that lncRNA-neighboring gene pairs were significantly more highly correlated than mRNA-neighboring gene pairs (Wilcoxon rank sum *p*-value < 2.2e-16). We pursued this result further. See Additional file 20 for details.

### LncRNA length, GC content, and antisense relative location

We only considered exonic sequence in lncRNA transcript length and GC content calculations. However, we included both intronic and exonic sequence in the antisense transcript location and annotated gene body length normalizations.

### LncRNA primer design

We designed PCR primers using AlleleID v7.6 software (Premier Biosoft International) to validate the exon structure of the five-exon Apicoplast RNA methyltransferase precursor antisense transcript, three-exon *ETRAMP* antisense transcript, and five-exon *PfGDV1* antisense transcript shown in Fig. 4. Except when noted in Additional file 25, we added a tail consisting of the T7 promoter sequence 5’-TAA TAC GAC TCA CTA TAG GG-3’ and 5’-TAG TAG TAG TAG-3’ to reverse primers, and a tail consisting of the M13F(−47) sequence 5’-CGC CAG GGT TTT CCC AGT CAC GAC-3’ and 5’-TAG TAG TAG TAG-3’ to forward primers. This allowed us to directly sequence PCR amplicons using universal T7 or M13F(−47) primers. Primer and expected amplicon sequences are listed in Additional file 25.

### LncRNA PCR and Sanger sequencing

We reverse transcribed 50 ng of total RNA from time course 2 time-points using SuperScript III (Invitrogen) and random hexamers (Invitrogen), and digested resulting cDNAs with 2 units of RNase H (Invitrogen). We then used the TaKaRa HotStart LA *Taq* polymerase (Clontech) and cDNA templates with the highest FPKM measurements for each targeted lncRNA [Additional files 16 and 25]. We cycled reactions as follows: 95 °C for 2 min, 35 cycles of 95 °C for 15 sec, 55 °C for 3 min, 60 °C for 3 min, 60 °C for 7 min, 65 °C for 7 min, 72 °C for 7 min. We gel-extracted products of the expected size at room temperature (Qiagen MinElute Kit), sequenced amplicons using GeneWiz services, and analyzed sequence chromatograms using Geneious v7.1.7 software (Biomatters) [108]. Amplicon size supported all 10 junctions, and Sanger sequencing read quality was sufficient in 9 cases. See Additional file 26 for diagrams showing Sanger sequencing read coverage and quality across targeted lncRNAs.

### CircRNA prediction

We predicted 1381 *P. falciparum* circRNAs using the methods developed by Memczak et al. [55]. In brief, we aligned reads to the *PlasmoDBv10.0* genome using Bowtie v2.1.0, and used circBase v1.0 scripts to split high-quality unaligned reads into anchors, subsequently screening anchors for linear or head-to-tail (circular) splicing. To get a reasonable set of circRNA candidates, we used the default circBase filtering suggestions, except for specifying a minimum circRNA size of 100 bp and a maximum circRNA size of 10,000 bp.

### Human microRNA binding site prediction in coding regions

For human microRNA binding site prediction in coding regions, we used the recently described PACCMIT-cds algorithm and the human microRNA v18 database (provided with the PACCMIT-cds distribution) [88]. The advantage of PACCMIT-cds is an improved background model that preserves both amino acid sequence and codon usage. We note, however, that the background model and significance calculations are not tuned for non-coding elements, and thus should be ignored or interpreted with caution in these cases. We first searched our six validated *P. falciparum* circRNA transcripts, and found that each candidate had predicted binding sites [Additional file 31]. Intrigued by this result, we then searched across *PlasmoDBv10.0* transcripts and found thousands of significant hits (10e6 precision) [Additional file 32].

### CircRNA expression correlation

To estimate whether the transcript levels of validated circRNAs tracked with the transcript levels of their respective linear mRNAs, we first multiplied the circRNA read counts per time-point by a library normalization factor. We defined the normalization factors as the maximum number of reads passing filtering in any time-point divided by the number of reads passing filtering in each time-point. We then computed the Pearson correlation between normalized circRNA read count profiles and respective linear mRNA log2(FPKM + 1) expression profiles [Column Q in Additional file 27]. As *P. falciparum* circRNAs appeared to be represented with far fewer reads than their linear mRNA counterparts, we were not concerned about circRNA read contributions to FPKM measurements.

### CircRNA primer design

We designed convergent and divergent PCR primers for nine *P. falciparum* genes with predicted circRNAs using AlleleID v7.6 software (Premier Biosoft International). Furthermore, we designed divergent forward primers as the reverse complement of convergent reverse primers, and divergent reverse primers as the reverse complement of convergent forward primers. We then added a tail consisting of the T7 promoter sequence 5’-TAA TAC GAC TCA CTA TAG GG-3’ and 5’-TAG TAG TAG TAG-3’ to all forward primers, and a tail consisting of the M13F(−47) sequence 5’-CGC CAG GGT TTT CCC AGT CAC GAC-3’ and 5’-TAG TAG TAG TAG-3’ to all reverse primers. This allowed us to directly sequence both divergent and convergent PCR amplicons using universal T7 or M13F(−47) primers. Primer and expected amplicon sequences are listed in Additional file 29.

### CircRNA PCR and Sanger sequencing

We reverse transcribed 50 ng of total RNA from time course 1 and time course 2 time-points using SuperScript III (Invitrogen) and random hexamers (Invitrogen), and digested resulting cDNAs with 2 units of RNase H (Invitrogen). We then used the KAPA HiFi HotStart polymerase (Kapa Biosystems) with cDNAs from time course 1, cDNAs from time course 2, *P. falciparum* gDNA (~30 ng), or water as the template. For each circRNA candidate, we tested two cDNAs (one from each time course) corresponding to the libraries with the maximum number of circRNA-specific read counts [Additional files 27 and 29]. We cycled convergent reactions as follows: 95 °C for 3 min, 35 cycles of 98 °C for 20 sec, 55 °C for 15 sec, ramp to 65 °C at 0.2 °C/sec, 65 °C for 15 sec, 65 °C for 7 min, 68 °C for 7 min, 72 °C for 7 min. We cycled divergent reactions equivalently, except that we performed an additional 5 cycles.

Convergent reactions yielded a product of the expected size (with positive Sanger sequencing) for 9/9 genes using time course 1 cDNAs, 8/9 genes using time course 2 cDNAs, and 7/9 genes using gDNA. Divergent reactions yielded a product of the expected size (with positive Sanger sequencing) for 6/9 genes using time course 1 cDNAs, 5/9 genes using time course 2 cDNAs, and 0/9 genes using gDNA. We gel-extracted products of the expected size at room temperature (Qiagen MinElute Kit), sequenced amplicons using GeneWiz services, and analyzed sequence chromatograms using Geneious v7.0.6 software (Biomatters) [108]. See Fig. 5D and Additional file 30 for divergent amplicon sequencing results.

## Availability of supporting data

The data set supporting the results of this article is available in the NCBI Gene Expression Omnibus repository [109], GSE57439 (http://www.ncbi.nlm.nih.gov/geo/query/acc.cgi?acc=GSE57439).

## Additional files

Additional file 1: **Library preparation flowchart.**

Additional file 2: **Library preparation protocol.**

Additional file 3: **Raw sequencing metrics.**

Additional file 4: **Aligned read metrics.**

Additional file 5: **Read quality box-plots.**

Additional file 6: ***PlasmoDBv10.0*** **transcript expression levels.**

Additional file 7: **GO term enrichment gene sets and results.**

Additional file 8: **Chimeric transcript predictions in the Cufflinks RABT assembly.**

Additional file 9: **Genome-guided Trinity assembly.**

Additional file 10: **Cufflinks annotation-free assembly.**

Additional file 11: **Cufflinks RABT assembly.**

Additional file 12: **Cufflinks assembled transcript property metrics.**

Additional file 13: **Cufflinks assembled transcript property plots.**

Additional file 14: **Comparison of bidirectional gene sets.**

Additional file 15: **Putative intergenic lncRNA properties.**

Additional file 16: **Putative antisense RNA properties.**

Additional file 17: **Novel and rediscovered transcript junctions.**

Additional file 18: **Transdecoder genome-wide coding region predictions.**

Additional file 19: **Upstream motif prediction details (unzip the folder and view the index.html file in a web browser).**

Additional file 20: **Additional neighboring gene correlation and distance plots.**

Additional file 21: **Antisense transcription initiation and termination locations.**

Additional file 22: ***PfGDV1*** **expression in the biological replicate time course.**

Additional file 23: **Chromosome ten left’s subtelomeric expression.**

Additional file 24: ***var*** **gene expression profiles.**

Additional file 25: **Library preparation and lncRNA PCR primer sequences.**

Additional file 26: **lncRNA structural validation results.**

Additional file 27: **Putative circRNAs.**

Additional file 28: **circRNA GO term enrichment results.**

Additional file 29: **circRNA PCR primers and expected amplicons.**

Additional file 30: **circRNA structural validation results.**

Additional file 31: **Predicted human microRNA binding sites across validated circRNAs.**

Additional file 32: **Predicted human microRNA binding sites across** ***PlasmoDBv10.0*** **transcripts.**

Additional file 33: **Genome-wide 100mer mappability track.**

Additional file 34: **Masked transcripts.**

Additional file 35: **Differentially expressed Cufflinks RABT genes.**

Additional file 36: **Alternatively spliced Cufflinks RABT transcripts.**

Additional file 37: **Alternative promoter usage cases in Cufflinks RABT assembly.**

Additional file 38: **Maximum sense-antisense pair expression.**

Additional file 39: **Periodicity in differentially expressed annotated mRNA profiles.**

Additional file 40: **Transcript specificity plots.**

## Abbreviations

lncRNAs: Long non-coding RNAs
RBC: Red blood cell
CVGE: Clonally variant gene expression
PfEMP1: *P. falciparum* Erythrocyte Membrane Protein 1
CLAG3: Cytoadherence Linked Asexual Gene 3
RISC: RNA-induced silencing complex
circRNA: circular RNA
hpi: hours post-infection
bp: base-pair
CV: Coefficient of variation
MDS: Multidimenstional scaling
mRNA: messenger RNA
RABT: Reference-annotation based transcript assembly
FPKM: Fragments per kilobase of exon per million fragments mapped
UTR: Untranslated region
ETRAMP: Early Transcribed Membrane Protein
PfGDV1: *P. falciparum* Gametocyte Development Protein 1
ARP: Apoptosis-related protein
cDNA: complementary DNA
gDNA: genomic DNA
MCA2: Metacaspase-like protein
PNA: Peptide nucleic acid
FSS: First strand synthesis
SSS: Second strand synthesis
USER: Uracil-Specific Excision Reagent
PF: Passing Illumina filtering
JS: Jensen-Shannon
TARE: Telomere-associated repetitive element
