# Supplementary material for: Strand-specific RNA sequencing in Plasmodium falciparum malaria identifies developmentally regulated long non-coding RNA and circular RNA
Source: BMC Genomics. 2015 Jun 13;16(1):454. doi: 10.1186/s12864-015-1603-4 (PMC4465157; doi:10.1186/s12864-015-1603-4)
Supplement: Supplementary file 1 — Library preparation flowchart. [file 12864_2015_1603_MOESM1_ESM.pdf]

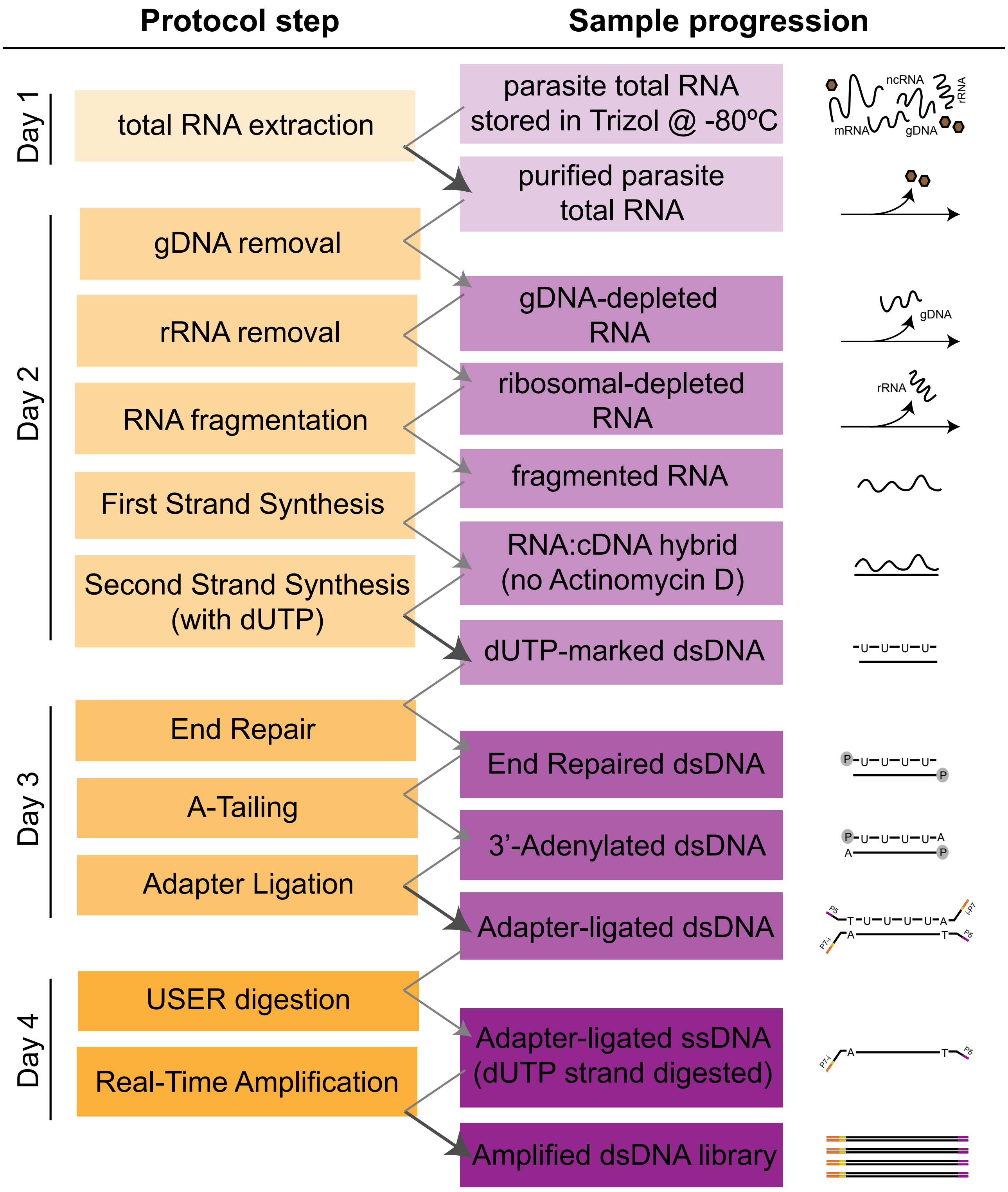

Figure S1. Strand-specific, non-polyA-selected *P. falciparum* libraries can be comfortably prepared in one week. The flowchart shows major protocol steps and sample progression during library preparation, beginning with parasite total RNA stored in TRIZOL Reagent and ending with amplified double-stranded DNA libraries ready for quantification and Illumina sequencing.
