# Supplementary material for: Strand-specific RNA sequencing in Plasmodium falciparum malaria identifies developmentally regulated long non-coding RNA and circular RNA
Source: BMC Genomics. 2015 Jun 13;16(1):454. doi: 10.1186/s12864-015-1603-4 (PMC4465157; doi:10.1186/s12864-015-1603-4)
Supplement: Supplementary file 2 — Library preparation protocol. [file 12864_2015_1603_MOESM2_ESM.docx]

**1. DNASE TREATMENT**

Reagents:

1. Up to 42 uL total RNA
2. Ambion TURBO DNase (order new lot, or ensure < 6 months old)
3. 10X TURBO DNase Buffer
4. Ambion SUPERase-In (20 U/uL)
5. Invitrogen RNaseOUT (40 U/uL)
6. Agencourt RNAclean SPRI beads
7. *Freshly prepared* 75% Ethanol
8. (optional) Agilent Bioanalyzer RNA 6000 Pico Kit

Protocol:

**Decontaminate work space, work quickly, and maintain RNase-free**

1. Combine the following in a PCR tube-strip:

| ___ uL | total RNA |
| --- | --- |
| ___ uL | H_2_0 |
| 5 uL | 10X TURBO DNase Buffer |
| 1 uL | SUPERase-In |
| 1 uL | RNaseOUT |
| 1 uL | TURBO DNase |
| 50 uL |  |

1. Incubate at room temperature for 30 minutes and proceed immediately to clean up to stop reaction
2. **1.8X RNAclean** SPRI bead purification in 96-well plate
   1. Equilibrate beads to room temperature for 30 minutes, vortex bead slurry thoroughly before adding to RNA and pipette beads slowly to avoid forming air bubbles
   2. Transfer 50 uL reaction to 96-well plate, add **90 uL beads**, and immediately pipette mixture 10 times upon bead addition
   3. Seal plate and incubate at room temperature for 20 minutes
   4. Separate beads on magnet for 5 minutes or until solution appears clear
   5. Remove and discard supernatant
   6. Keeping plate on magnetic rack, wash the beads in 200 uL freshly prepared 75% Ethanol for at least 30 seconds
   7. Carefully remove and discard ethanol without disturbing pellet and repeat the process for a total of **2 washes**
   8. Allow beads to air dry for 10 minutes (ensure no ethanol traces remain on sides of wells)
   9. Resuspend beads in **30 uL h20** and incubate at room temperature for 5 minutes
   10. Separate beads on magnet for 5 minutes or until solution appears clear
   11. Carefully transfer 26-28 uL fragmented RNA to new tube/plate
3. Proceed immediately to Ribosomal RNA depletion
4. *Recommended:* save 1 uL aliquot of RNA to check RNA quality using an Agilent Bioanalyzer RNA 6000 Pico Kit

**2. RIBOSOMAL RNA DEPLETION**

Reagents:

1. 1-5 ug DNase-treated RNA (cleaned up, in RNase-free h20)
   1. 26 uL maximum volume (for 2.5-5 ug) or 28 uL for < 2.5 ug input
2. Epicentre Ribo-Zero Magnetic Kit (Human/Mouse/Rat)
3. Heating block for 1.5 mL tubes, **set to 50⁰C**
4. Agencourt RNAclean SPRI beads
5. *Freshly prepared* 75% Ethanol
6. (optional) Agilent Bioanalyzer RNA 6000 Pico Kit

Protocol:

**Decontaminate work space, work quickly, and maintain RNase-free**

1. Equilibrate Ribo-Zero Magnetic Core Kit components to room temperature for 30 minutes
2. Remove the Ribo-Zero rRNA Removal Kit from -80⁰C, thaw the tubes, and place on ice
3. Individual Magnetic Bead washing procedure:
   1. Mix the Ribo-Zero Magnetic Beads by gentle vortexing
   2. For each reaction, *slowly* pipette 225 uL of Ribo-Zero Magnetic Beads into a 1.5 mL RNase-free tube. Store unused beads at 4⁰C
   3. Separate beads on magnet for 5 minutes or until solution appears clear
   4. With the tube still on the stand, remove and discard the supernatant
   5. Remove the tube from stand and add 225 uL of RNase-free h20. Mix well by vortexing at medium speed
   6. Repeat steps *c* and *d* and remove the tube from the magnetic stand
   7. Add 65 uL of Ribo-Zero Magnetic Bead Suspension Solution to each tube. Mix well by vortexing at medium speed
   8. Add 1 uL of RiboGuard RNase Inhibitor to each tube of resuspended Magnetic Beads. Mix briefly by vortexing.
   9. Store at room temperature until required in step *5*
4. Treatment of total RNA with Ribo-Zero rRNA Removal Solution
   1. In a PCR tube-strip combine the following:

| _____ uL | h20 |
| --- | --- |
| 4 uL | Ribo-Zero Reaction Buffer |
| _____ uL | 2.5-5 ug total RNA sample* |
| 10 uL | Ribo-Zero rRNA Removal Solution |
| 40 uL |  |

*If < 2.5 ug total RNA, adjust Ribo-Zero rRNA Removal Solution to 8 uL

1. Gently mix the reaction(s) by pipetting and incubate at 68⁰C for 10 minutes
2. Store the remaining Ribo-Zero rRNA Removal Solution and Reaction Buffer at -80⁰C
3. Remove the reaction tubes and incubate at room temperature for 5 minutes.
4. Magnetic Bead Reaction and rRNA Removal
   1. Using a pipette, add the treated RNA from step *4* to the 1.5 mL tube containing the washed Magnetic Beads and immediately mix by pipetting at least 10 times. Then, vortex at medium setting for 10 seconds and place at room temperature
   2. Incubate the 1.5 mL tube at room temperature for 5 minutes
   3. Following incubation, mix the reations by vortexing at medium speed for 5 seconds and then place tube in heat block set to **50⁰C for exactly 5 minutes.**
   4. Immediately transfer tube to magnetic stand and allow beads to separate for 5 minutes or until solution appears clear
   5. Carefully remove each supernatant (~90 uL) containing the RNA and transfer to a NEW RNase-free tube
      1. The supernatant contains the rRNA-depleted RNA
   6. Place the supernatant (RNA solution) on ice and immediately proceed to cleanup
5. **1.8X RNAclean** SPRI bead purification
   1. Equilibrate beads to room temperature for 30 minutes, vortex bead slurry thoroughly before adding to RNA and pipette beads slowly to avoid forming air bubbles
   2. In 1.5mL tube combine 90uL RNA + **162 uL beads**, immediately pipette mixture 10 times upon bead addition
   3. Close cap and incubate at room temperature for 20 minutes
   4. Separate beads on magnet for 5 minutes or until solution appears clear
   5. Remove and discard supernatant
   6. Keeping tubes on magnetic rack, wash the beads in 500 uL freshly prepared 75% Ethanol for at least 30 seconds
   7. Carefully remove and discard ethanol without disturbing pellet and repeat the process for a total of **2 washes**
   8. Allow beads to air dry for 10 minutes (ensure no ethanol traces remain on inside of tube)
   9. Resuspend beads in **21 uL h20** and incubate at room temperature for 5 minutes
   10. Separate beads on magnet for 5 minutes or until solution appears clear
   11. Carefully transfer 18 uL Ribo-Zero’d RNA to new tube/plate
6. Proceed immediately to RNA fragmentation
7. *Recommended:* save 1 uL aliquot of Ribo-Zero’d RNA to check ribosomal RNA depletion using an Agilent Bioanalyzer RNA Pico 6000 kit

**3. RNA FRAGMENTATION**

Reagents:

1. 18 uL DNase-treated, rRNA-depleted RNA (cleaned up, in h20)
2. NEB Mg^2+^ Fragmentation Buffer
3. Agencourt RNAclean SPRI beads
4. *Freshly prepared* 75% Ethanol
5. (optional) Agilent Bioanalyzer RNA 6000 Pico Kit

Protocol:

**Decontaminate work space, work quickly, and maintain RNase-free techniques**

1. Combine in PCR-tube strip:

| 18 uL | DNase’d RNA |
| --- | --- |
| 2 uL | NEB Mg^2+^ Frag Buffer |
| 20 uL |  |

1. Incubate in PTC-225 DNA Engine Tetrad (MJ Research) thermocycler at 85⁰C for 8 minutes (YES heated lid) and transfer immediately to ice
   1. 8 minutes yields fragments 100-1000 bp in length; 300 bp average. Increasing time will shift distribution towards smaller fragments.
   2. Place in thermocycler exactly as lid reaches 85⁰C
   3. Remove and place on ice exactly when begins to lower temperature
   4. Proceed immediately to cleanup to STOP reaction
2. **1.8X RNAclean** SPRI bead purification
   1. Equilibrate beads to room temperature for 30 minutes, vortex bead slurry thoroughly before adding to RNA and pipette beads slowly to avoid forming air bubbles
   2. Transfer 20 uL reaction to 96-well plate, add **36 uL beads**, and immediately pipette mixture 10 times upon bead addition
   3. Seal plate and incubate at room temperature for 20 minutes
   4. Separate beads on magnet for 5 minutes or until solution appears clear
   5. Remove and discard supernatant
   6. Keeping plate on magnetic rack, wash the beads in 200 uL freshly prepared 75% Ethanol for at least 30 seconds
   7. Carefully remove and discard ethanol without disturbing pellet and repeat the process for a total of **2 washes**
   8. Allow beads to air dry for 10 minutes (ensure no ethanol traces remain on sides of wells)
   9. Resuspend beads in **9 uL h20** and incubate at room temperature for 5 minutes
   10. Separate beads on magnet for 5 minutes or until solution appears clear
   11. Carefully transfer 7 uL Fragmented RNA to new tube/plate
3. Proceed immediately to First-Strand Synthesis (FSS)
4. *Recommended:* save 1 uL aliquot of RNA to check fragmentation using an Agilent Bioanalyzer RNA Pico 6000 Kit

**4. First Strand Synthesis (FSS)**

Reagents:

1. 7 uL Fragmented RNA (cleaned-up, in h20)
2. Invitrogen 5X FS buffer
3. 3 ug/uL random primers ***see Special Notes section***
4. 10 mM dNTP mix
5. 100 mM DTT
6. Invitrogen Superscript III
7. Invitrogen RNaseOUT (40 U/uL)
8. Actinomycin D solution (1 ug/uL) ***see Special Notes section***
9. Agencourt RNAclean SPRI beads
10. Bio-Rad Micro Bio-Spin P-30 columns, Tris buffer (RNase-free)
11. *Freshly prepared* 75% Ethanol
12. (optional) Agilent Bioanalyzer RNA 6000 Pico Kit

Protocol:

1. Combine the following in a PCR tube-strip:

| 7 uL | Fragmented RNA |
| --- | --- |
| 1 uL | 3 ug/uL custom random primers |
| 1 uL | 10mM dNTP mix |
| 9 uL |  |

1. Gently vortex and spin down
2. Incubate at 65⁰C for 5 min in a PTC-225 DNA Engine Tetrad (MJ Research) thermocycler and transfer immediately to ice for 5 minutes
3. Add the following components to the reaction on ice:

| 4 uL | 5X FS Buffer |
| --- | --- |
| 1 uL | 100 mM DTT |
| 4 uL | 1 ug/uL Actinomycin D solution |
| 1 uL | RNaseOUT |
| 1 uL | Superscript III (200 U) |
| 11 uL |  |

1. Incubate in PTC-225 DNA Engine Tetrad (MJ Research) thermocycler:
   1. Set ramp speed for each transition to .1⁰C/second, YES heated lid
      1. 5⁰C – 5 minutes
      2. 10⁰C – 5 minutes
      3. 15⁰C – 5 minutes
      4. 20⁰C – 5 minutes
      5. 25⁰C – 5 minutes
      6. 30⁰C – 5 minutes
      7. 35⁰C – 5 minutes
      8. 42⁰C – 30 minutes 🡨 optional: @ 30” + 1uL SSIII (200 U)
      9. 45⁰C – 10 minutes
      10. 50⁰C – 10 minutes
      11. 55⁰C – 10 minutes
      12. Hold at 4⁰C forever
      13. **DO NOT HEAT KILL ENZYME**
2. Raise reaction volume to 50 uL by adding 30 uL h20
3. Clean-up with Micro Bio-Spin P-30 RNase-free column to remove dNTPs/ActD
   1. Obtain columns from 4⁰C cold room (check that < 1yr old)
   2. Forcefully invert column 10 times whilst flicking to remove air bubbles
   3. Snap off fret and remove cap
   4. Let column drain for 3 minutes or until visibly drained
   5. Dump drained buffer and spin in supplied collection tube at 1000 x g for 2 minutes
   6. Place column in a new RNase-free collection tube (labeled appropriately)
   7. Load 50 uL sample to center of gel bed without disturbing it
   8. Spin at 1000 x g for 4 minutes (orient tubes as in previous spin)
   9. Sample is now in 10 mM Tris-HCl, pH 7.4
4. **1.8X RNAclean** SPRI bead purification to remove primers (+dNTPs/ActD)
   1. Equilibrate beads to room temperature for 30 minutes, vortex bead slurry thoroughly before adding to RNA and pipette beads slowly to avoid forming air bubbles
   2. Check that ~45 uL reaction remains in 1.5 mL tube, add **~80 uL beads**, and immediately pipette mixture 10 times upon bead addition
   3. Close cap and incubate at room temperature for 30 minutes
   4. Separate beads on magnet for 5 minutes or until solution appears clear
   5. Remove and discard supernatant
   6. Keeping plate on magnetic rack, wash the beads in 500 uL freshly prepared 75% Ethanol for at least 30 seconds
   7. Carefully remove and discard ethanol without disturbing pellet and repeat the process for a total of **2 washes**
   8. Allow beads to air dry for 10 minutes (ensure no ethanol traces remain on sides of wells)
   9. Resuspend beads in **95 uL h20** and incubate at room temperature for 5 minutes
   10. Separate beads on magnet for 5 minutes or until solution appears clear
   11. Carefully transfer 88 uL RNA:cDNA hybrid to new tube/plate
5. Proceed immediately to Second-Strand Synthesis (SSS)
6. *Recommended:* save 1 uL aliquot of RNA:cDNA hybrid to check yield/size distribution/primer removal using an Agilent Bioanalyzer RNA Pico 6000 Kit
7. *Recommended:* save remainder of RNA:cDNA hybrid (~5 uL) for qPCR QC check
   1. Check genomic DNA removal using (–) enzyme control

**5. Second Strand Synthesis**

Reagents:

1. 88 uL RNA:cDNA hybrid (cleaned up 2X, in h20)
2. Invitrogen 5X FS Buffer
3. 100 mM DTT
4. Fermentas 2mM dACG-TP/4mM dU-TP mix
5. Invitrogen 5X SS Buffer
6. *E. coli* DNA ligase (10U/uL)
7. *E. coli* DNA polymerase (10U/uL)
8. *E. coli* RNase H (2U/uL)
9. 0.5 M EDTA
10. Agencourt AmpureXP SPRI beads
11. Qiagen Buffer EB
12. *Freshly prepared* 75% Ethanol
13. (optional) Agilent Bioanalyzer High Sensitivity DNA Kit

Protocol:

1. Combine the following in a PCR tube-strip:

| 88 uL | cDNA:RNA hybrid |
| --- | --- |
| 4 uL | 5X FS Buffer |
| 2 uL | 100 mM DTT |
| 20 uL | 2 mM DACG-TP/dU-TP mix |
| 30 uL | 5X SS Buffer |
| 1 uL | *E. coli* DNA ligase (10 U) |
| 4 uL | *E. coli* DNA polymerase (40 U) |
| 1 uL | *E. coli* RNase H (2 U) |
| 150 uL (+62 uL Master Mix) |  |

1. Mix gently by pipetting and incubate at **15⁰C** in PTC-225 DNA Engine Tetrad (MJ Research) thermocycler for 2 hours
   1. **Do not vortex**
   2. NO HEATED LID Do not let sample temperature raise above 16⁰C or DNA polymerase can strand displace
   3. Transfer immediately to ice
2. Add 10 uL 0.5M EDTA to STOP reaction and proceed immediately to cleanup
3. **1.8X AmpureXP** SPRI bead purification
   1. Equilibrate beads to room temperature for 30 minutes, vortex bead slurry thoroughly before adding to RNA and pipette beads slowly to avoid forming air bubbles
   2. Transfer ~155 uL reaction to 1.5mL tube, add **~280 uL beads**, and immediately pipette mixture 10 times upon bead addition
   3. Close cap and incubate at room temperature for 10 minutes
   4. Separate beads on magnet for 5 minutes or until solution appears clear
   5. Remove and discard supernatant
   6. Keeping plate on magnetic rack, wash the beads in 500 uL freshly prepared 75% Ethanol for at least 30 seconds
   7. Carefully remove and discard ethanol without disturbing pellet and repeat the process for a total of **2 washes**
   8. Allow beads to air dry for 10 minutes (ensure no ethanol traces remain on sides of wells)
   9. Resuspend beads in **88 uL Qiagen EB** and incubate at room temperature for 5 minutes
   10. Separate beads on magnet for 5 minutes or until solution appears clear
   11. Carefully transfer 85 uL dUTP-marked dsDNA to new tube/plate
4. ***SAFE STOPPING POINT***
   1. Proceed immediately to KAPA End Repair ***OR*** store dUTP-marked dsDNA at -20⁰C for up to 1 week
5. *Recommended:* save 1 uL aliquot of dUTP-marked dsDNA to check yield/size distribution using an Agilent Bioanalyzer HS DNA chip

**6. KAPA End Repair**

Reagents:

1. 85 uL dUTP-marked dsDNA (cleaned-up, in EB)
2. KAPA library prep kit
3. Agencourt AmpureXP SPRI beads
4. Qiagen Buffer EB
5. *Freshly prepared* 75% Ethanol

Protocol:

1. Combine the following in a PCR tube strip:

| 85 uL | dUTP-marked dsDNA |
| --- | --- |
| 10 uL | 10X KAPA End Repair Buffer |
| 5 uL | KAPA End Repair Enzyme Mix* |
| 100 uL |  |

*15U T4 DNA Polymerase, 50U T4 Polynucleotide Kinase

1. Mix gently and incubate for 30 minutes at 20⁰C in PTC-225 DNA Engine Tetrad (MJ Research) thermocycler
   1. No heated lid
   2. Transfer to ice and proceed immediately to cleanup
2. **1.8X AmpureXP** SPRI bead purification
   1. Equilibrate beads to room temperature for 30 minutes, vortex bead slurry thoroughly before adding to RNA and pipette beads slowly to avoid forming air bubbles
   2. Transfer 100uL reaction to 1.5mL tube, add **180 uL beads**, immediately pipette mixture 10 times upon bead addition
   3. Close cap and incubate at room temperature for 10 minutes
   4. Separate beads on magnet for 5 minutes or until solution appears clear
   5. Remove and discard supernatant
   6. Keeping tubes on magnetic rack, wash the beads in 500 uL freshly prepared 75% Ethanol for at least 30 seconds
   7. Carefully remove and discard ethanol without disturbing pellet and repeat the process for a total of **2 washes**
   8. Allow beads to air dry for 10 minutes (ensure no ethanol traces remain on inside of tube)
   9. Resuspend beads in **33 uL Qiagen EB** and incubate at room temperature for 5 minutes
   10. Separate beads on magnet for 5 minutes or until solution appears clear
   11. Carefully transfer 31 uL End-Repaired dsDNA to new tube/plate
3. ***SAFE STOPPING POINT***
   1. Proceed immediately to KAPA A-Tailing ***OR*** store End-Repaired dsDNA at -20⁰C for up to 1 week

**7. KAPA A-Tailing**

Reagents:

1. 30 uL End-Repaired dsDNA (cleaned-up, in EB)
2. KAPA library prep kit
3. Agencourt AmpureXP SPRI beads
4. Qiagen Buffer EB
5. *Freshly prepared* 75% Ethanol

Protocol:

1. Combine the following in a PCR tube strip:

| 30 uL | End-Repaired dsDNA |
| --- | --- |
| 12 uL | H_2_0 |
| 5 uL | 10X KAPA A-Tailing Buffer* |
| 3 uL | KAPA A-Tailing Enzyme** |
| 50 uL |  |

*contains 2 mM dATP (.2 mM final)

**15U 3’🡪5’ exo^-^ klenow fragment

1. Mix gently and incubate for 30 minutes at 30⁰C in PTC-225 DNA Engine Tetrad (MJ Research) thermocycler
   1. No heated lid
   2. Transfer to ice and proceed immediately to cleanup
2. **1.8X AmpureXP** SPRI bead purification
   1. Equilibrate beads to room temperature for 30 minutes, vortex bead slurry thoroughly before adding to RNA and pipette beads slowly to avoid forming air bubbles
   2. Add **90 uL beads**, immediately pipette mixture 10 times upon bead addition
   3. Close cap and incubate at room temperature for 10 minutes
   4. Separate beads on magnet for 5 minutes or until solution appears clear
   5. Remove and discard supernatant
   6. Keeping tubes on magnetic rack, wash the beads in 500 uL freshly prepared 75% Ethanol for at least 30 seconds
   7. Carefully remove and discard ethanol without disturbing pellet and repeat the process for a total of **2 washes**
   8. Allow beads to air dry for 10 minutes (ensure no ethanol traces remain on inside of tube)
   9. Resuspend beads in **33.5 uL Qiagen EB and** incubate at room temperature for 5 minutes
   10. Separate beads on magnet for 5 minutes or until solution appears clear
   11. Carefully transfer 31 uL A-Tailed dsDNA to new tube/plate
3. ***SAFE STOPPING POINT***
   1. Proceed immediately to KAPA Adapter Ligation ***OR*** store A-Tailed dsDNA at -20⁰C for up to 1 week

**8. KAPA Adapter Ligation**

Reagents:

1. 30 uL A-Tailed dsDNA (cleaned-up, in EB)
2. KAPA library prep kit
3. 15 uM Barcoded Y-Adapters
4. Agencourt AmpureXP SPRI beads
5. Qiagen Buffer EB
6. *Freshly prepared* 75% Ethanol

Protocol:

**Change gloves/clean pipette every time handle different adapter**

1. Dilute adapters approximately 1:10 in h20 (1.5 uM final). See calculation below.
2. Combine the following in a PCR tube strip:

| 30 uL | A-Tailed dsDNA |
| --- | --- |
| 10 uL | 5X KAPA Ligation Buffer* |
| 5 uL | 1.5 uM Barcoded Y-Adapter **#______**_______ |
| 5 uL | KAPA Ligation Enzyme Mix** |
| 50 uL |  |

*Contains 30% PEG 6000 (6% final). Raising PEG concentration lowers SPRI bead size selection (i.e. smaller fragments elute off beads than normal)

**30,000U T4 DNA Ligase

***Calculate molar ratio of dsDNA target:Adapter to ensure in the 1:15 regime. Remember that each dsDNA fragment represents 2 targets for adapter ligation but that ER/A-tailing is not 100% efficient…. If assume 50% efficiency these scaling factors cancel each other out. Example calculation:

| (30 ng A-Tailed dsDNA, 300 bp)*(1 mole/300bp*650g) = 1.54 * 10^-4^ nmoles |
| --- |
| (5uL * 1.5 uM Adapters)*(L/10^6^ uL) = 2.5 * 10^-3^ nmoles |
| **~1:15** |

1. Mix gently and incubate for 15 minutes at 20⁰C in PTC-225 DNA Engine Tetrad (MJ Research) thermocycler
   1. No heated lid
   2. Transfer immediately to ice
2. Proceed immediately to SPRI Bead Adapter removal

**9. SPRI Bead Adapter Removal**

Reagents:

1. 50 uL Adapter-Ligated dsDNA
2. Agencourt AmpureXP SPRI beads
3. Qiagen Buffer EB
4. *Freshly prepared* 75% Ethanol
5. (optional) Agilent Bioanalyzer High Sensitivity DNA Kit

Protocol:

1. **1.0X AmpureXP** SPRI bead purification
   1. Equilibrate beads to room temperature for 30 minutes, vortex bead slurry thoroughly before adding to RNA and pipette beads slowly to avoid forming air bubbles
   2. Add **50 uL beads**, immediately pipette mixture 10 times upon bead addition
   3. Close cap and incubate at room temperature for 15 minutes
   4. Separate beads on magnet for 5 minutes or until solution appears clear
   5. Remove and discard supernatant
   6. Keeping tubes on magnetic rack, wash the beads in 500 uL freshly prepared 75% Ethanol for at least 30 seconds
   7. Carefully remove and discard ethanol without disturbing pellet and repeat the process for a total of **2 washes**
   8. Allow beads to air dry for 15 minutes (ensure no ethanol traces remain on inside of tube)
   9. Resuspend beads in **22 uL Qiagen EB and** incubate at room temperature for 5 minutes
   10. Separate beads on magnet for 5 minutes or until solution appears clear
   11. Carefully transfer 20 uL Adapter-Ligated dsDNA to new tube/plate
2. ***SAFE STOPPING POINT***
   1. Proceed immediately to USER digestion ***OR*** store Adapter-Ligated dsDNA at -20⁰C for up to 1 week
3. *Recommended:* save 1 uL aliquot of Adapter-Ligated dsDNA to check yield/size distribution/adapter dimer contamination using an Agilent Bioanalyzer HS DNA chip.

**10. USER digestion (DO NOT FORGET TO DIGEST dUTP-marked strand)**

Reagents:

1. 47 uL Adapter-ligated dsDNA (cleaned up 1X or 2X)
2. NEB USER (Uracil-Specific Excision Reagent) enzyme (1U/uL)
3. (optional) Agilent Bioanalyzer RNA Pico Kit
4. (optional) Agilent Bioanalyzer High Sensitivity DNA Kit

Protocol:

1. Add 1 uL USER enzyme to each reaction
2. Incubate at 37⁰C for 30 minutes, followed by 25⁰C for 15 minutes
   1. USER is not heat inactivated
   2. Yes heated lid
3. ***SAFE STOPPING POINT***
   1. Proceed immediately to KAPA Real-Time PCR Amplification ***OR*** store USER-digested ssDNA at -20⁰C
4. *Recommended:* save 1 uL aliquot of USER-digested ssDNA to check yield/size distribution/adapter dimer contamination using an Agilent Bioanalyzer RNA Pico 6000 Kit
5. *Recommended:* save 1 uL aliquot of USER-digested ssDNA to check second strand digestion using an Agilent Bioanalyzer HS DNA chip

**11. KAPA Real-Time PCR Amplification**

Reagents:

1. 9 uL USER-digested ssDNA (~1/5 reaction used as input)
2. KAPA real-time library amplification kit
3. 12.5 uM *each* Forward and Reverse Indexing PCR Primer Mix
4. Agencourt AmpureXP SPRI beads
5. Qiagen Buffer EB
6. *Freshly prepared* 75% Ethanol
7. Agilent Bioanalyzer High Sensitivity DNA Kit

Protocol:

1. Add 65uL of fluorescence references 1-4 to a 96-well plate
2. Combine the following for each library in 96-well plate:

| 9 uL | ssDNA sample (USER-digested) |
| --- | --- |
| 2 uL | F&R Indexing PCR Primer Mix* |
| 11 uL | 2X KAPA HiFi SYBR/Rox qPCR Mix |
| 22 uL |  |

*1 uM each (final)

1. Seal, vortex and spin down plate
2. Transfer 3 X 20 uL of each reference to a 384-well plate using multi-channel pipette
3. Add 20 uL of each library to 384-well plate
4. Seal and spin down plate
   1. Ensure no debris or air bubbles
5. Thermocycle on ABI 7900 Real-Time PCR Machine with following program
   1. 98⁰C – 2 minutes
   2. 20 cycles:
      1. 98⁰C – 20 seconds
      2. **55⁰C* – 180 seconds**

*Optimal annealing at 55⁰C for BROAD indexing PCR primers

- - 1. 55⁰C – 10 seconds **DATA ACQUISITION**

1. SIT IN ROOM AND WATCH THE PCR MACHINE THE ENTIRE TIME TO STOP REACTION WHEN LIBRARY BETWEEN REFERENCE 1 and 3
   1. Must stop during data acquisition phase before machine ramps to 98⁰C
   2. The less PCR cycles the better. Do not over-cycle.
   3. If expect vastly different yields, amplify libraries individually or perform a diagnostic amplification and repeat with normalized sample input.
2. **1.0X AmpureXP** SPRI bead purification
3. Equilibrate beads to room temperature for 30 minutes, vortex bead slurry thoroughly before adding to RNA and pipette beads slowly to avoid forming air bubbles
4. Transfer 20uL reaction to 96-well plate, add 20uL beads, immediately pipette mixture 10 times upon bead addition
5. Seal plate and incubate at room temperature for 10 minutes
6. Separate beads on magnet for 5 minutes or until solution appears clear
7. Remove and discard supernatant
8. Keeping plate on magnetic rack, wash the beads in 200 uL freshly prepared 75% Ethanol for at least 30 seconds
9. Carefully remove and discard ethanol without disturbing pellet and repeat the process for a total of **2 washes**
10. Allow beads to air dry for 10 minutes (ensure no ethanol traces remain on inside of tube)
11. Resuspend beads in **25 uL Qiagen EB and** incubate at room temperature for 5 minutes
12. Separate beads on magnet for 5 minutes or until solution appears clear
13. Carefully transfer 22.5 uL PCR-amplified dsDNA to new tube/plate
14. ***SAFE STOPPING POINT***
    1. Proceed immediately to KAPA Library Quantification ***OR*** store PCR-amplified dsDNA at -20⁰C
15. *Recommended:* save 1 uL aliquot of PCR-amplified final library to check yield/size distribution/adapter dimer contamination using an Agilent Bioanalyzer HS DNA chip. If necessary, repeat 1.0X AmpureXP SPRI bead purification to further remove adapter dimers.

***Special Notes***

*Ideally, custom order 76% AT-biased random hexamers to reflect the approximate AT balance of the *P. falciparum* transcriptome.

*Prepare the Actinomycin D solution freshly in water, keep it protected from light, and handle it with caution, as ActD is highly toxic. Verify the concentration of ActD solution using Beer’s law immediately prior to use. Chilling the ActD solution may increase solubility (!).
