# Supplementary material for: Strand-specific RNA sequencing in Plasmodium falciparum malaria identifies developmentally regulated long non-coding RNA and circular RNA
Source: BMC Genomics. 2015 Jun 13;16(1):454. doi: 10.1186/s12864-015-1603-4 (PMC4465157; doi:10.1186/s12864-015-1603-4)
Supplement: Supplementary file 5 — Read quality box-plots. [file 12864_2015_1603_MOESM5_ESM.pdf]

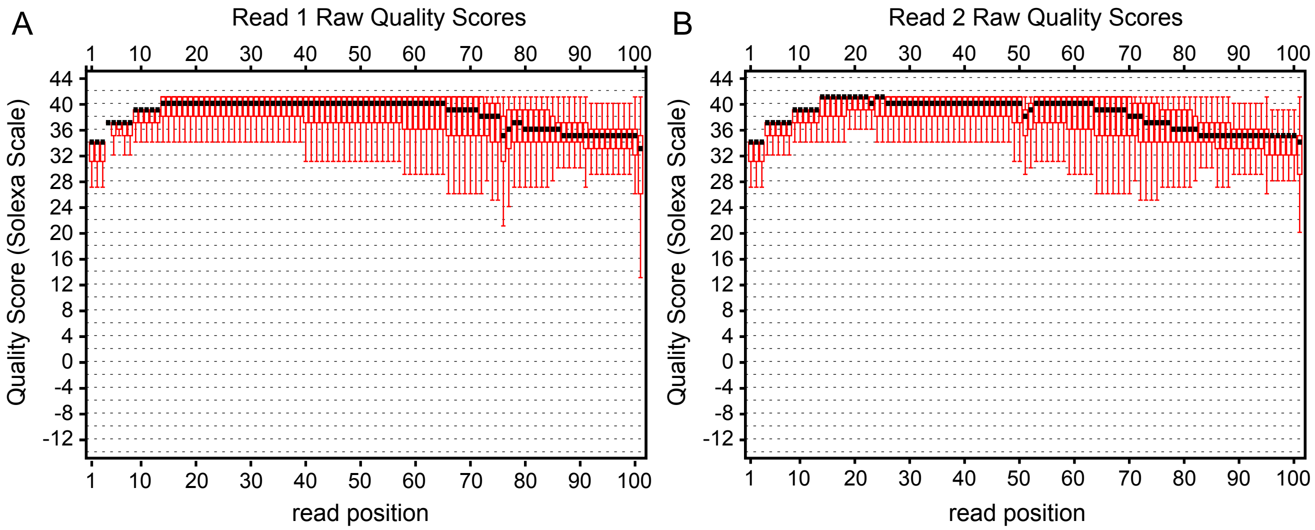

Figure S5. Raw reads have high quality scores. (A/B) Read 1 and Read 2 raw quality scores, respectively. Each plot summarizes approximately 307 million reads agglomerated from all fifteen samples. We trimmed the last base and filtered the 4-8% of reads from each sample that did not pass Illumina filtering prior to read alignment.
