# Supplementary material for: Strand-specific RNA sequencing in Plasmodium falciparum malaria identifies developmentally regulated long non-coding RNA and circular RNA
Source: BMC Genomics. 2015 Jun 13;16(1):454. doi: 10.1186/s12864-015-1603-4 (PMC4465157; doi:10.1186/s12864-015-1603-4)
Supplement: Supplementary file 13 — Cufflinks assembled transcript property plots. [file 12864_2015_1603_MOESM13_ESM.pdf]

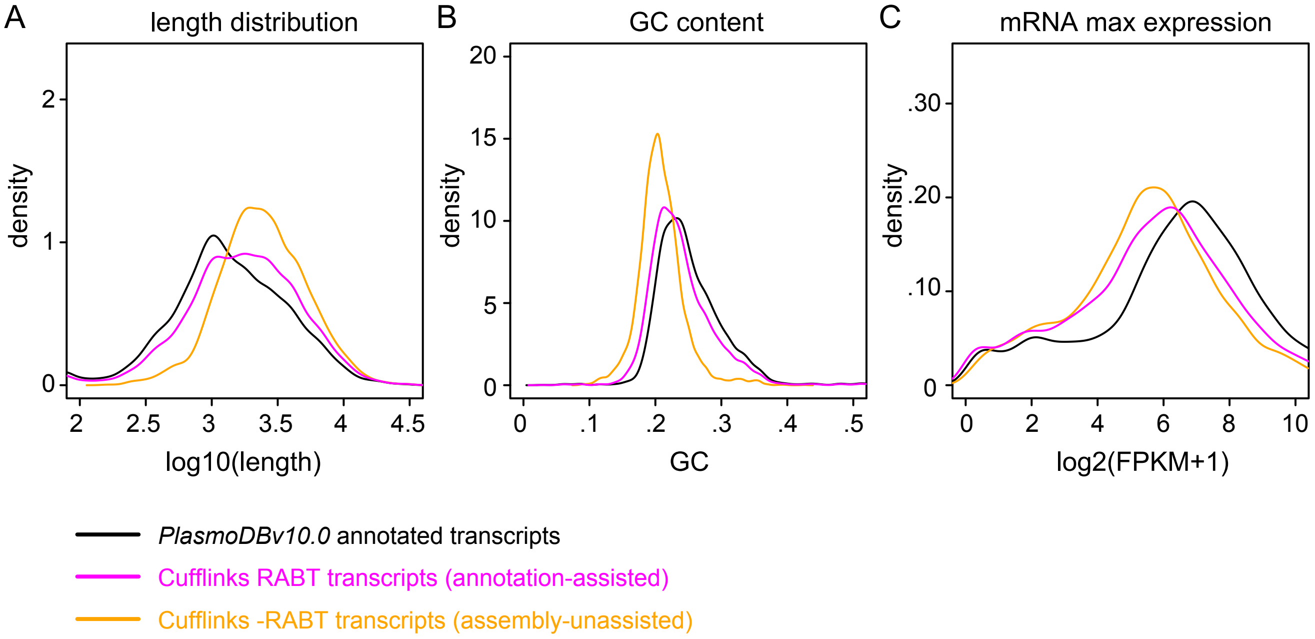

Figure S13. Cufflinks assembled transcripts tend to include un-annotated regions, likely to be UTRs. Upon comparing the properties of *PlasmoDBv10.0* annotated transcripts (black), Cufflinks RABT transcripts (pink), and Cufflinks -RABT transcripts (orange), we found evidence for un-annotated, potential UTRs included in the Cufflinks assembled transcripts. This was supported by the slightly (A) increased length, and (B) reduced GC content of Cufflinks assembled transcripts, as *P. falciparum* non-coding regions typically have lower GC content than coding regions. Also, when we considered mRNA transcript expression levels during the 56-hour time course, we found (C) reduced maximum expression levels of Cufflinks assembled mRNA transcripts, which further indicated that Cufflinks transcripts included UTRs with less read coverage.
