# Supplementary material for: Strand-specific RNA sequencing in Plasmodium falciparum malaria identifies developmentally regulated long non-coding RNA and circular RNA
Source: BMC Genomics. 2015 Jun 13;16(1):454. doi: 10.1186/s12864-015-1603-4 (PMC4465157; doi:10.1186/s12864-015-1603-4)
Supplement: Supplementary file 14 — Comparison of bidirectional gene sets. [file 12864_2015_1603_MOESM14_ESM.pdf]

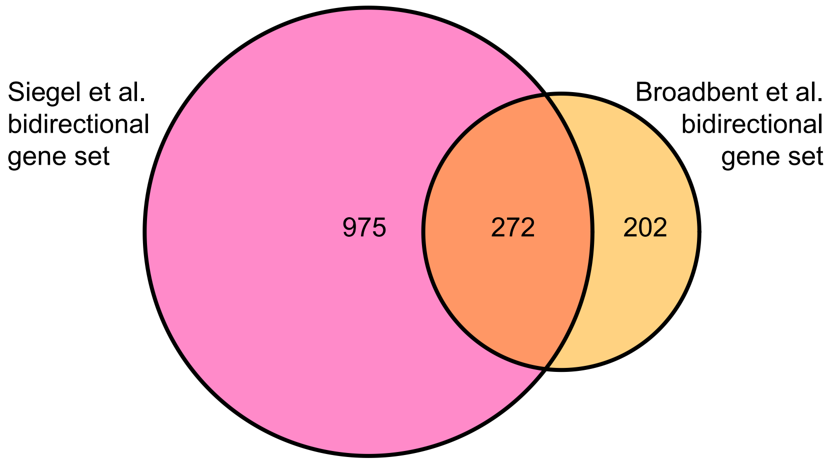

Figure S14. Overlap with a previously described bidirectional gene set. Comparing our bidirectional (sense and antisense producing) gene set to the Siegel et al. bidirectional gene set indicated significant differences [1]. The Siegel et al. study included only four time-points, which could account for the 202 novel genes found here. The Siegel et al. study also did not involve assembly of transcript structures, and we required assembled transcript structures to be supported by at least fifty (perfectly and uniquely) mapped reads in a single sample. Our use of different methods and a higher threshold for expression may account for the 975 genes predicted by Siegel et al. only. Indeed, we calculated that the average maximum RPKM of “stringent” antisense regions reported by Siegel et al. was 4, while the average maximum FPKM of our antisense transcripts was 25. The FPKM (fragment) measurements reported by Cufflinks are analgous to single-end RPKM (read) measurements [2].

1. Siegel TN, Hon CC, Zhang Q, Lopez-Rubio JJ, Scheidig-Benatar C, et al. (2014) Strand-specific RNA-Seq reveals widespread and developmentally regulated transcription of natural antisense transcripts in Plasmodium falciparum. BMC Genomics 15: 150.

2. Mortazavi A, Williams BA, McCue K, Schaeffer L, Wold B (2008) Mapping and quantifying mammalian transcriptomes by RNA-Seq. Nat Methods 5: 621-628.
