# Supplementary material for: Strand-specific RNA sequencing in Plasmodium falciparum malaria identifies developmentally regulated long non-coding RNA and circular RNA
Source: BMC Genomics. 2015 Jun 13;16(1):454. doi: 10.1186/s12864-015-1603-4 (PMC4465157; doi:10.1186/s12864-015-1603-4)
Supplement: Supplementary file 20 — Additional neighboring gene correlation and distance plots. [file 12864_2015_1603_MOESM20_ESM.pdf]

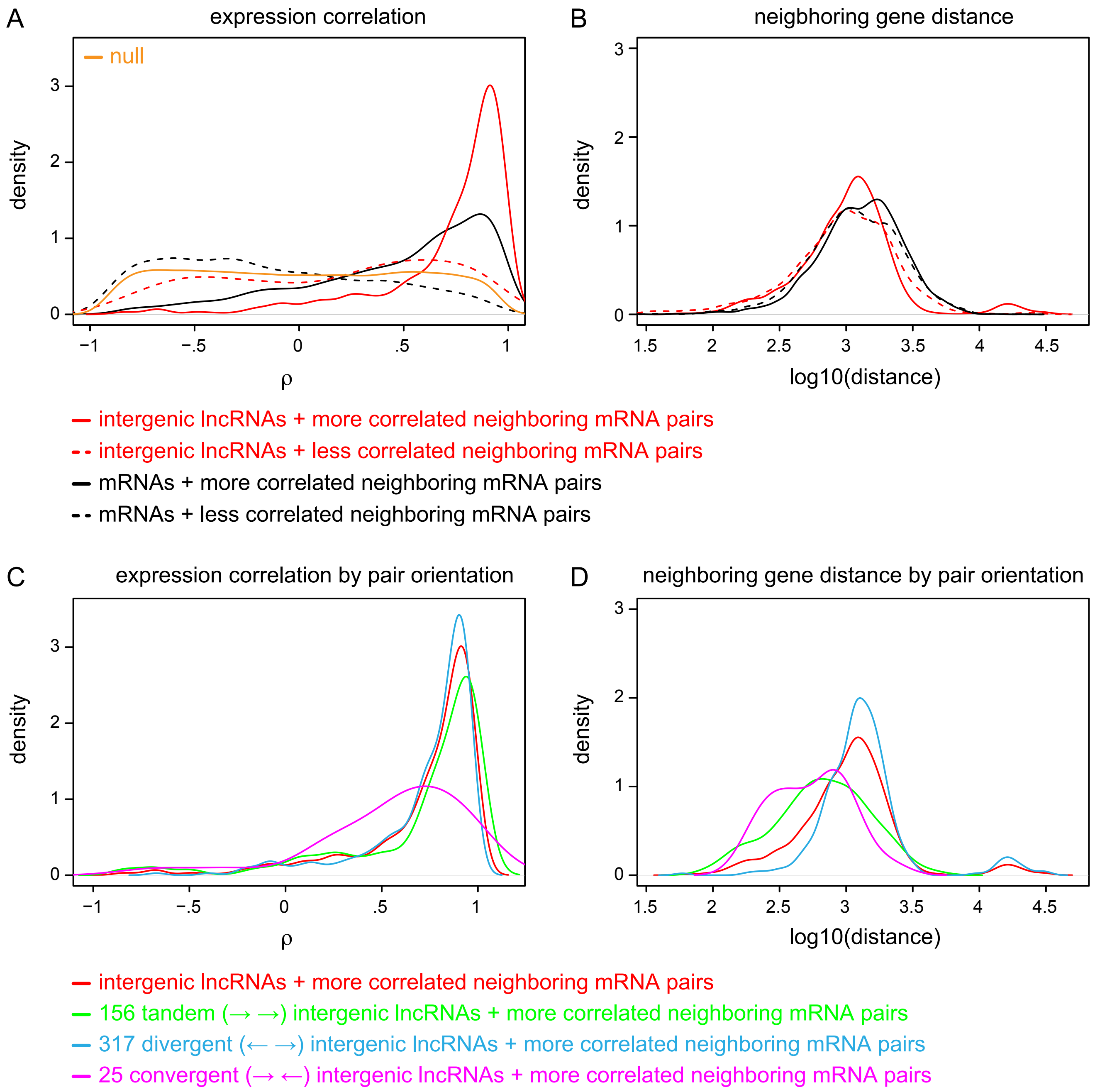

Figure S20. **Two-dimensional genomic distance between neighboring loci is not a strong indicator of expression correlation.** (A)/(B) The Pearson correlation during the 56-hour time course and the distance between mRNA-neighbor pairs (black) and intergenic lncRNA-neighbor pairs (red), respectively. Dashed lines correspond to pairings with the less correlated neighbor. This analysis indicated that both mRNAs and intergenic lncRNAs had a significantly more correlated neighbor. However, regardless of pairing criteria, the genomic distances between neighboring loci were quite similar. (C/D) We next stratified the Pearson correlation during the 56-hour time-course and the distance between intergenic lncRNAs and their more correlated neighbor by pair orientation, respectively. We found 156 tandem, 317 divergent, and 25 convergent pairs. Notably, convergent loci, albeit exhibiting less extreme positive correlation, were not more distant from each other.
