# Supplementary material for: Strand-specific RNA sequencing in Plasmodium falciparum malaria identifies developmentally regulated long non-coding RNA and circular RNA
Source: BMC Genomics. 2015 Jun 13;16(1):454. doi: 10.1186/s12864-015-1603-4 (PMC4465157; doi:10.1186/s12864-015-1603-4)
Supplement: Supplementary file 21 — Antisense transcription initiation and termination locations. [file 12864_2015_1603_MOESM21_ESM.pdf]

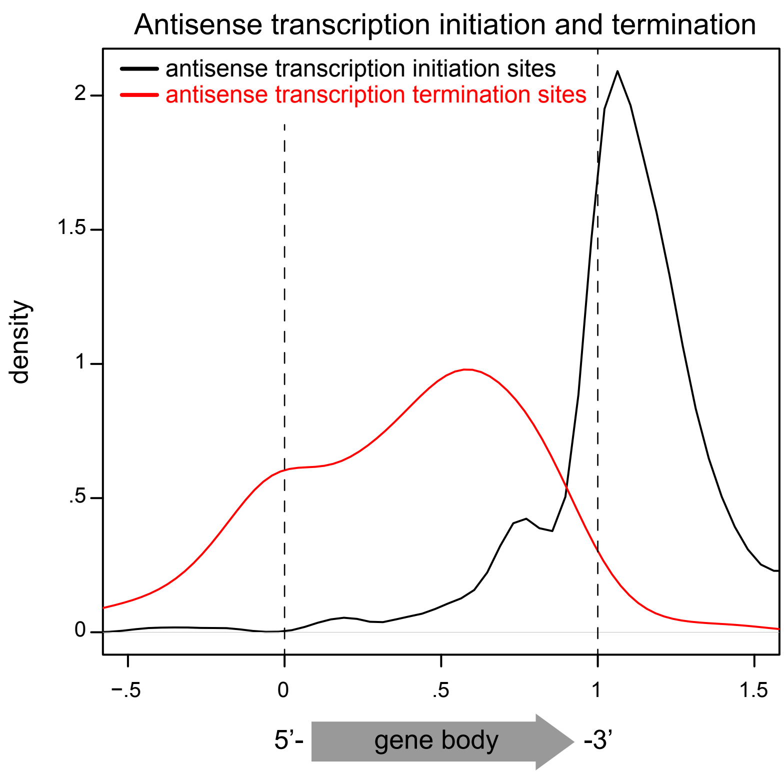

Figure S21. Sense-antisense transcripts tend to overlap in their 3' tail region. The distribution of antisense transcription initiation sites (black) and antisense transcription termination sites (red) showed that the vast majority of antisense transcripts initiated transcription downstream of gene bodies, and tended to terminate transcription towards the 3' tail region as well. We normalized antisense transcription initiation and termination sites to the location and length of their respective *PlasmoDBv10.0* annotated gene partner, inclusive of intronic sequence.
