# Supplementary material for: Strand-specific RNA sequencing in Plasmodium falciparum malaria identifies developmentally regulated long non-coding RNA and circular RNA
Source: BMC Genomics. 2015 Jun 13;16(1):454. doi: 10.1186/s12864-015-1603-4 (PMC4465157; doi:10.1186/s12864-015-1603-4)
Supplement: Supplementary file 22 — PfGDV1 expression in the biological replicate time course. [file 12864_2015_1603_MOESM22_ESM.pdf]

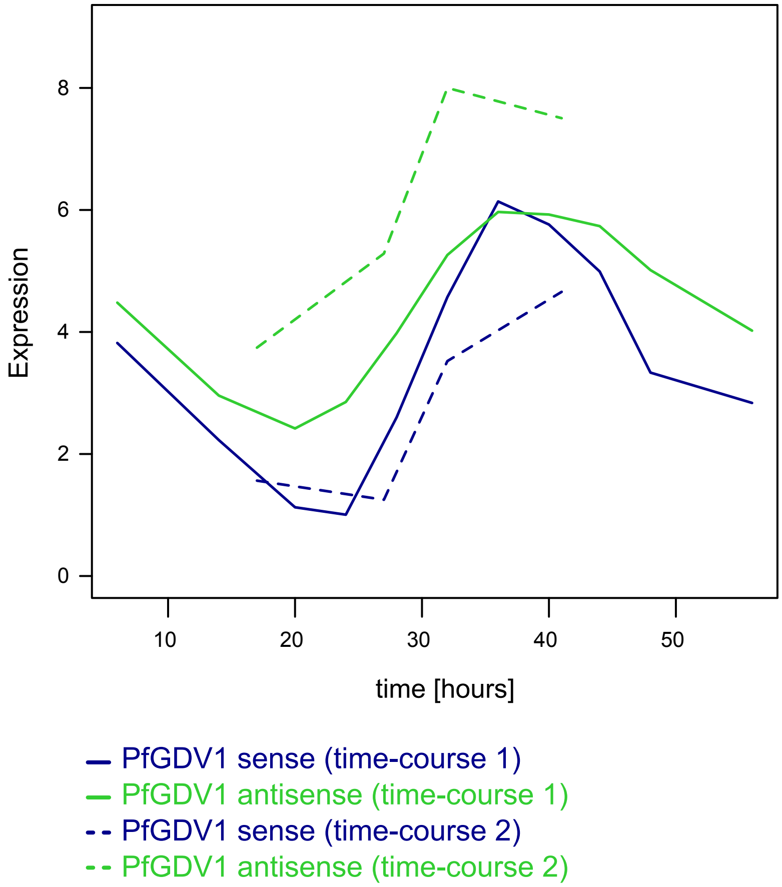

Figure S22. Comparison of *PfGDV1* antisense transcript levels in two time courses. Plotting *PfGDV1* expression during both time-courses showed that *PfGDV1* sense-antisense transcript levels were highly correlated. Antisense transcript levels during the shorter time course reached a maximum FPKM of 255. Pearson correlation during the shorter time course was 0.85. We estimated the shorter time course time-points from our MDS staging analysis as approximately: 17, 27, 32, and 41 hpi. Expression is plotted in units of log2(FPKM+1).
