# Supplementary material for: Strand-specific RNA sequencing in Plasmodium falciparum malaria identifies developmentally regulated long non-coding RNA and circular RNA
Source: BMC Genomics. 2015 Jun 13;16(1):454. doi: 10.1186/s12864-015-1603-4 (PMC4465157; doi:10.1186/s12864-015-1603-4)
Supplement: Supplementary file 23 — Chromosome ten left’s subtelomeric expression. [file 12864_2015_1603_MOESM23_ESM.pdf]

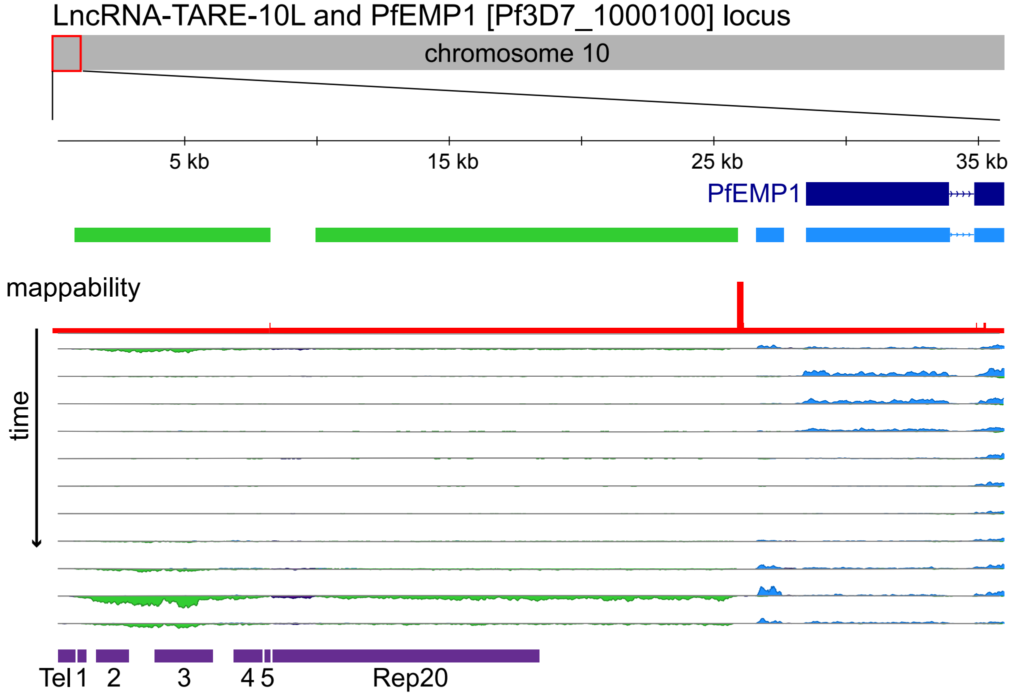

Figure S23. Transcription of the entire chromosome ten left subtelomere during invasion. Normalized read alignment tracks across the subtelomeric upsB-type PfEMP1 encoding *var* gene (*Pf3D7_1000100*) and chromosome ten left subtelomeric TARE region. The annotated *Pf3D7_1000100* gene model is shown in dark blue, and assembled transcript models are shown in light green and light blue. Reads from each 56-hour time course sample mapping to the (-) strand are shown below each horizontal axis in light green, while reads mapping to the (+) strand are shown above each horizontal axis in light blue. Intron reads are shown in purple. Uniqueness of 100mers is plotted in red as a mappability track. Boundaries of the telomere, TAREs 1-5, and Rep20 are shown in purple as well.
