# Supplementary material for: Strand-specific RNA sequencing in Plasmodium falciparum malaria identifies developmentally regulated long non-coding RNA and circular RNA
Source: BMC Genomics. 2015 Jun 13;16(1):454. doi: 10.1186/s12864-015-1603-4 (PMC4465157; doi:10.1186/s12864-015-1603-4)
Supplement: Supplementary file 24 — var gene expression profiles. [file 12864_2015_1603_MOESM24_ESM.pdf]

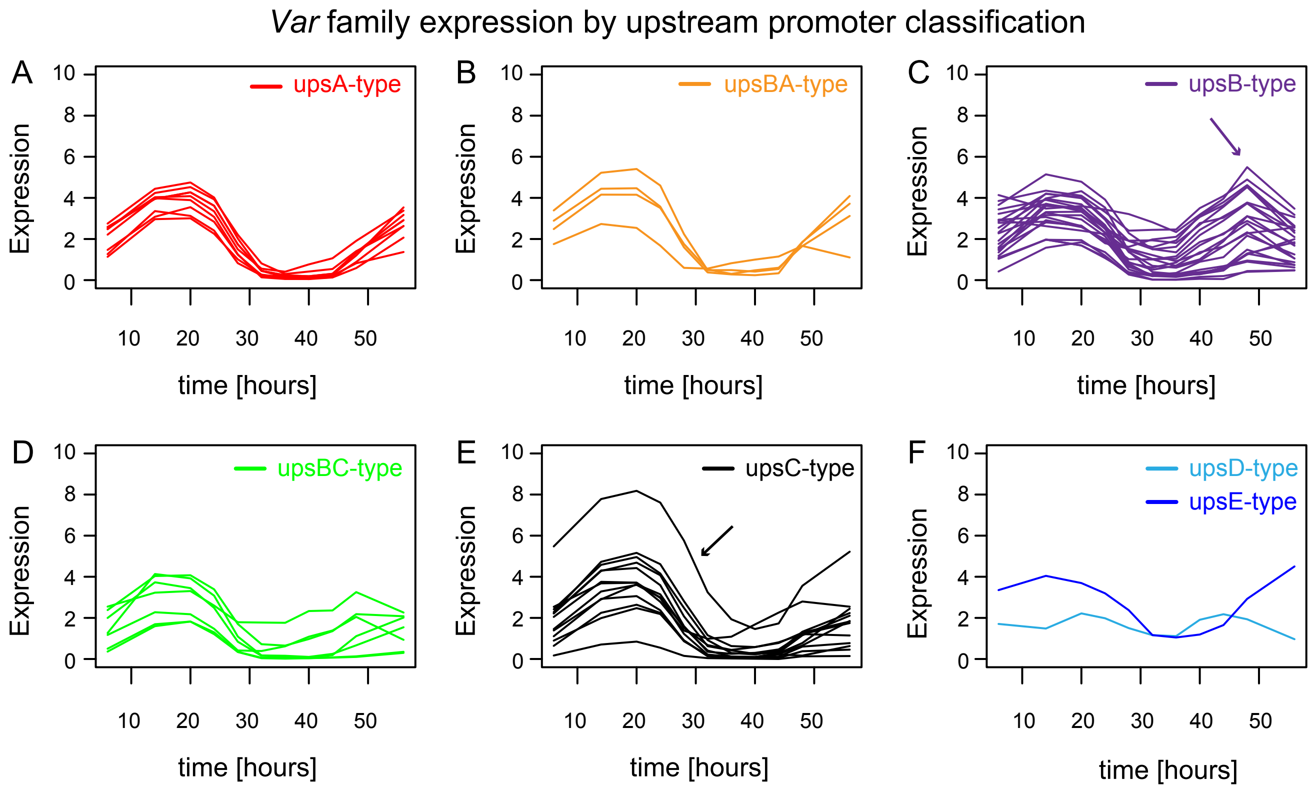


**Figure S24. A central upsC-type *var* gene is the dominant *var* transcript, and subtelomeric upsB-type *var* genes produce the most non-coding transcripts.** (A/B/C/D/E/F) Expression during the 56-hour time course of upsA-type (red), upsBA-type (orange), upsB-type (purple), upsBC-type (green), upsC-type (black), upsD-type (light blue) and upsE-type (dark blue) *var* genes, respectively [1]. This analysis indicated that an upsC-type gene, *Pf3D7_0412700*, was the dominant *var* transcript in the parasite population (black arrow). We also noted that the transcription of non-coding transcripts varied by upstream promoter (ups) type. Interestingly, the majority of upsB-type *var* genes transcribed non-coding transcripts during parasite invasion (purple arrow), whereas the majority of upsA-type and upsC-type *var* genes did not. Expression is plotted in units of log2(FPKM+1). upsA-type: *Pf3D7_0425800, Pf3D7_1150400, Pf3D7_1300300, Pf3D7_0800200, Pf3D7_1100200, Pf3D7_0400400, Pf3D7_0100300, Pf3D7_0937600, Pf3D7_0600400*; upsBA-type: *Pf3D7_0800300, Pf3D7_1200400, Pf3D7_0600200, Pf3D7_0632500*; upsB-type: *Pf3D7_1100100, Pf3D7_0800100, Pf3D7_0500100, Pf3D7_0100100, Pf3D7_0115700, Pf3D7_0324900, Pf3D7_0400100, Pf3D7_0900100, Pf3D7_0223500, Pf3D7_1041300, Pf3D7_1200100, Pf3D7_0200100, Pf3D7_0300100, Pf3D7_1255200, Pf3D7_0426000, Pf3D7_0937800, Pf3D7_1000100, Pf3D7_1219300, Pf3D7_0632800, Pf3D7_1373500, Pf3D7_0733000, Pf3D7_0700100, Pf3D7_1300100, Pf3D7_0833500*; upsBC-type: *Pf3D7_0413100, Pf3D7_1240400, Pf3D7_0808700, Pf3D7_0712800, Pf3D7_0712400, Pf3D7_0421100, Pf3D7_1240300*; upsC-type: *Pf3D7_0712900, Pf3D7_0808600, Pf3D7_0420700, Pf3D7_0712000, Pf3D7_0412900, Pf3D7_0420900, Pf3D7_0421300, Pf3D7_0412400, Pf3D7_0712600, Pf3D7_0711700, Pf3D7_1240600, Pf3D7_0412700, Pf3D7_0617400*; upsD-type: *Pf3D7_0533100*; upsE-type: *Pf3D7_1200600.*

1. Lavstsen T, Salanti A, Jensen AT, Arnot DE, Theander TG (2003) Sub-grouping of Plasmodium falciparum 3D7 var genes based on sequence analysis of coding and non-coding regions. Malaria Journal 2: 27.
