# Supplementary material for: Strand-specific RNA sequencing in Plasmodium falciparum malaria identifies developmentally regulated long non-coding RNA and circular RNA
Source: BMC Genomics. 2015 Jun 13;16(1):454. doi: 10.1186/s12864-015-1603-4 (PMC4465157; doi:10.1186/s12864-015-1603-4)
Supplement: Supplementary file 26 — lncRNA structural validation results. [file 12864_2015_1603_MOESM26_ESM.pdf]

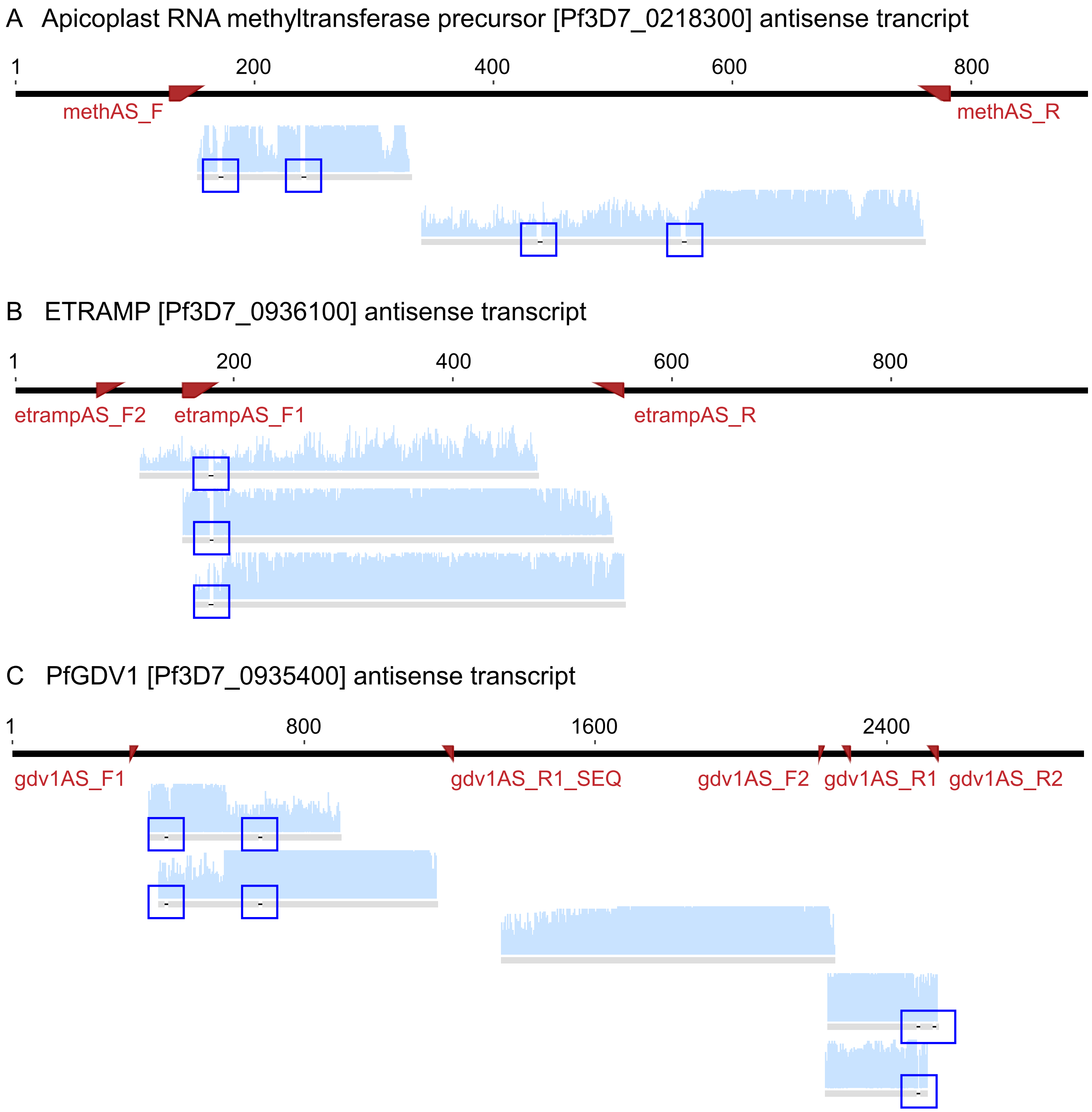

Figure S26. Experimental confirmation of multi-exonic *P. falciparum* lncRNAs. (A)/(B)/(C) PCR primer locations and Sanger sequencing read coverage across amplicons, for multi-exonic lncRNA transcripts predicted antisense to Pf3D7_0218300, Pf3D7_0936100, and Pf3D7_0935400, respectively. Blue boxes highlight predicted splice junctions validated by Sanger sequencing.
