# Supplementary material for: Strand-specific RNA sequencing in Plasmodium falciparum malaria identifies developmentally regulated long non-coding RNA and circular RNA
Source: BMC Genomics. 2015 Jun 13;16(1):454. doi: 10.1186/s12864-015-1603-4 (PMC4465157; doi:10.1186/s12864-015-1603-4)
Supplement: Supplementary file 30 — circRNA structural validation results. [file 12864_2015_1603_MOESM30_ESM.pdf]

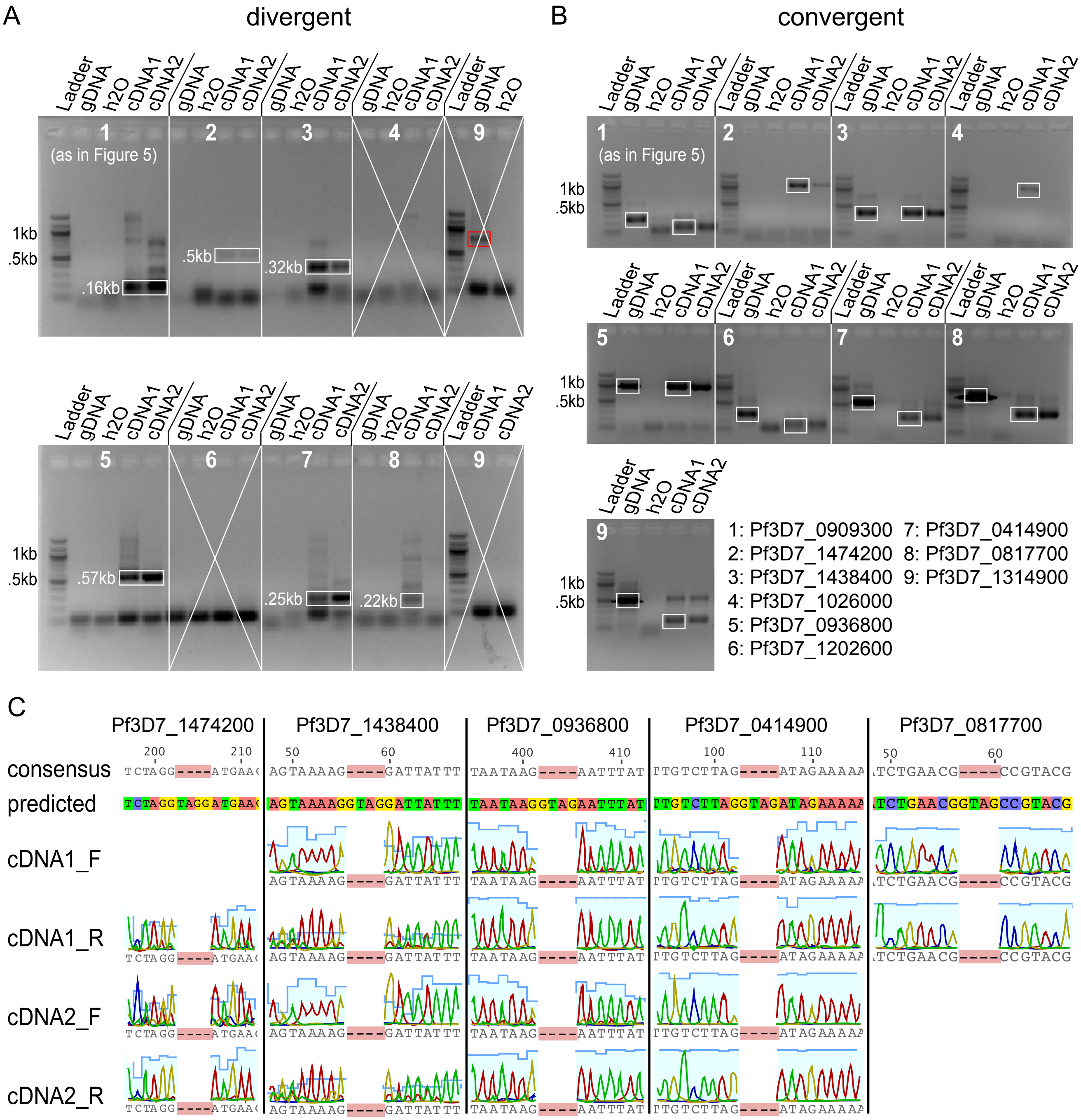

Figure S30. Experimental confirmation of five additional *P. falciparum* circRNAs. (A)/(B) Divergent and convergent PCR reactions, respectively, for the nine tested loci using gDNA, water (h20), and cDNA prepared from either time-course (cDNA1/2). Ladder and divergent amplicon sizes are indicated. We gel-excised and sequenced each boxed amplicon. White boxes indicate amplicons of the expected size with positive Sanger sequencing confirmation. The red box indicates a non-specific amplicon (according to sequencing results). Smaller amplicon sizes in the convergent cDNA reactions as compared to the convergent gDNA reactions corresponded to intron removal. The larger products in the divergent cDNA reactions may represent non-specific or rolling-circle reverse transcription products. (C) Sanger sequencing across five additional circRNA splice junctions.
