# Supplementary material for: Strand-specific RNA sequencing in Plasmodium falciparum malaria identifies developmentally regulated long non-coding RNA and circular RNA
Source: BMC Genomics. 2015 Jun 13;16(1):454. doi: 10.1186/s12864-015-1603-4 (PMC4465157; doi:10.1186/s12864-015-1603-4)
Supplement: Supplementary file 38 — Maximum sense-antisense pair expression. [file 12864_2015_1603_MOESM38_ESM.pdf]

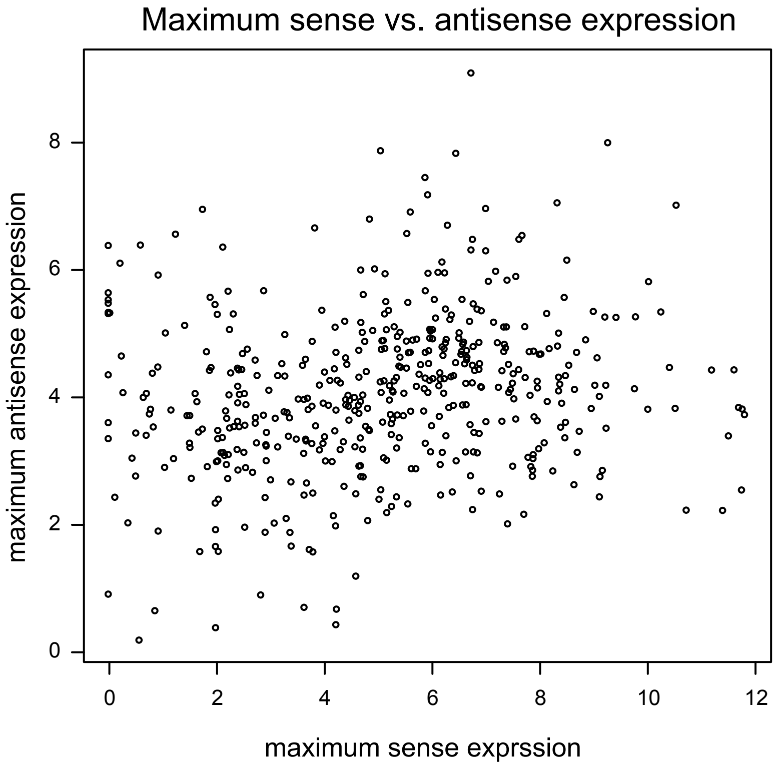


**Figure S38. Maximum antisense expression level does not strongly correlate with maximum sense expression level.** Each point represents a bidirectionally transcribed (sense and antisense) gene. We compared maximum sense and antisense transcript levels during the 56-hour time course. Expression is plotted in units of log2(FPKM+1).
