# Supplementary material for: Strand-specific RNA sequencing in Plasmodium falciparum malaria identifies developmentally regulated long non-coding RNA and circular RNA
Source: BMC Genomics. 2015 Jun 13;16(1):454. doi: 10.1186/s12864-015-1603-4 (PMC4465157; doi:10.1186/s12864-015-1603-4)
Supplement: Supplementary file 39 — Periodicity in differentially expressed annotated mRNA profiles. [file 12864_2015_1603_MOESM39_ESM.pdf]

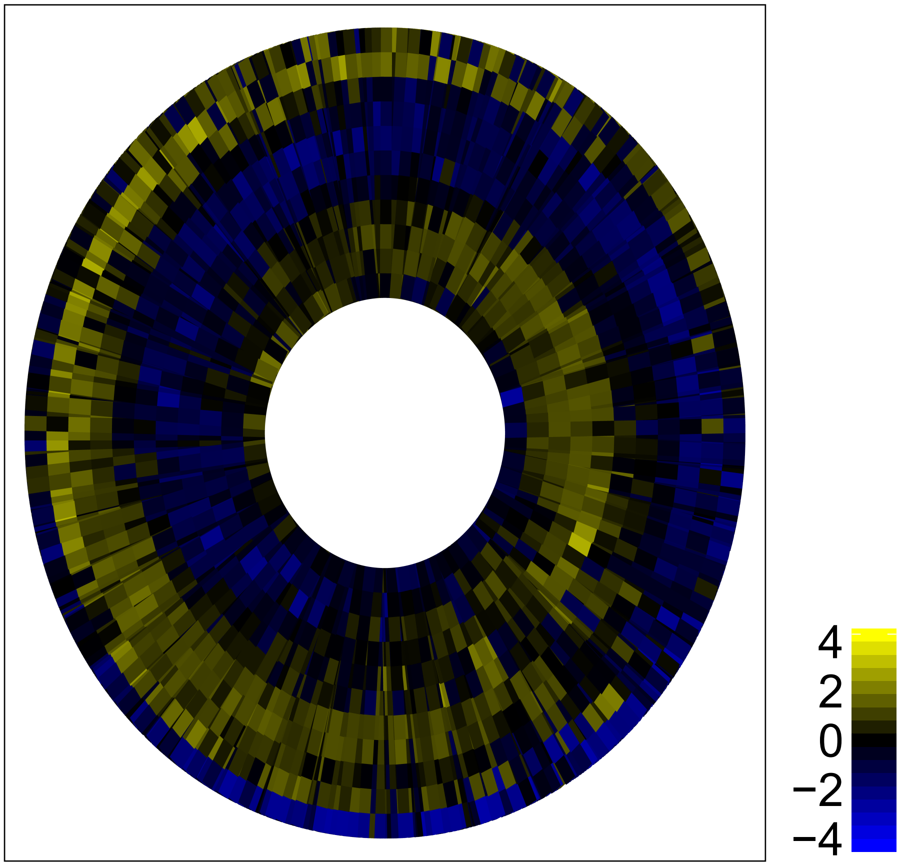

Figure S39. Periodicity is evident in *P. falciparum* blood stage expression profiles. For differentially expressed annotated mRNA genes, we visualized mean-centered expression profiles across the 56-hour time course in a circular, non-clustering heatmap. Genes are ordered by their angular position in the MDS plot of gene expression profiles and samples are ordered by time. Expression is in units of log2(FPKM+1).
