# Supplementary material for: Strand-specific RNA sequencing in Plasmodium falciparum malaria identifies developmentally regulated long non-coding RNA and circular RNA
Source: BMC Genomics. 2015 Jun 13;16(1):454. doi: 10.1186/s12864-015-1603-4 (PMC4465157; doi:10.1186/s12864-015-1603-4)
Supplement: Supplementary file 40 — Transcript specificity plots. [file 12864_2015_1603_MOESM40_ESM.pdf]

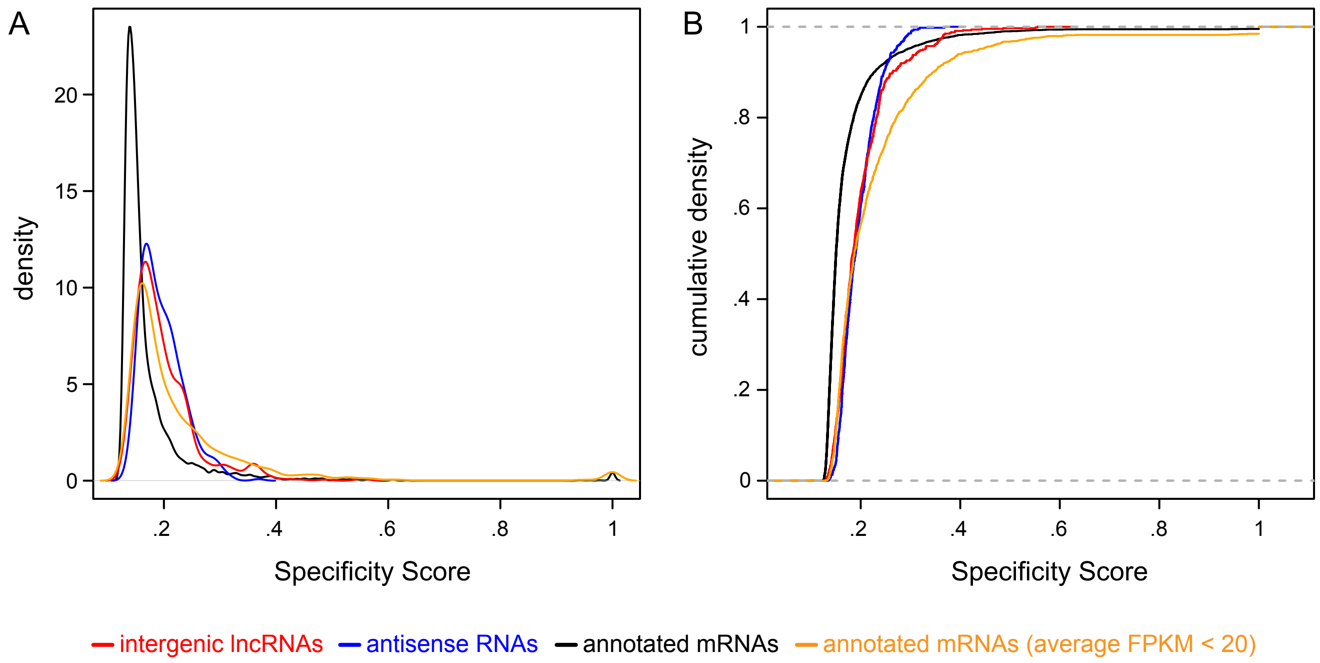

Figure S40. Stage-specificity of *P. falciparum* lncRNAs as compared to mRNAs. (A) The distribution of specificity scores for each transcript class indicated that intergenic lncRNAs (red) and antisense RNAs (blue) typically had higher specificity scores than annotated mRNAs (black). However, when we considered only lowly expressed mRNAs, we found that the 1619 annotated mRNAs with an average FPKM of less than 20 (orange) had a comparable distribution of specificity scores to lncRNAs. (B) As in panel A, except that the cumulative distribution of specificity scores is plotted.
